# Impact of phenolic compounds in the acyl homoserine lactone-mediated quorum sensing regulatory pathways

**DOI:** 10.1038/s41598-017-10997-5

**Published:** 2017-09-06

**Authors:** Md. Akil Hossain, Seung-Jin Lee, Na-Hye Park, Abraham Fikru Mechesso, Biruk Tesfaye Birhanu, JeongWoo Kang, Md. Ahsanur Reza, Joo-Won Suh, Seung-Chun Park

**Affiliations:** 1 Laboratory of Clinical Pharmacokinetics and Pharmacodynamics, College of Veterinary Medicine, Kyungpook National University, Daegu, 702-701 Republic of Korea; 2 Veterinary drugs & Biologics Division, Animal and Plant Quarantine Agency (QIA), 177, Hyeoksin 8-ro, Gimcheon-si, Gyeongsangbuk-do 39660 Republic of Korea; 3 Faculty of Animal Science and Veterinary Medicine, Patuakhali Science and Technology University (Outer Campus), Babugonj, Barisal 8210 Bangladesh; 4 Center for Nutraceutical and Pharmaceutical Materials, Division of Bioscience and Bioinformatics, Science campus, Myongji University, 449-728 Yongin, Gyeonggi Republic of Korea

## Abstract

Quorum sensing (QS) is a cell density-dependent regulation of virulent bacterial gene expression by autoinducers that potentially pertains in the epidemic of bacterial virulence. This study was initially designed to evaluate the effect of 5 phenolic compounds in the modulation of QS and virulence factors of *Chromobacterium violaceum* and *Pseudomonas aeruginosa*, and to determine the mechanisms of their effects. Biosensor strains were used to assess antibacterial and anti-QS effect of these compounds. Only methyl gallate (MG) among these compounds demonstrated profound anti-QS effect in the preliminary study, and thus only MG was utilized further to evaluate the effects on the synthesis and activity of acyl homoserine lactone (AHL) in *C. violaceum* and on the modulation of biofilm, motility, proteolytic, elastase, pyocyanin, and rhamnolipid activity in *P. aeruginosa*. Finally, the effect of MG on the expression of QS-regulated genes of *P. aeruginosa* was verified. MG suppressed both the synthesis and activity of AHL in *C. violaceum*. It also restricted the biofilm formation and other QS-associated virulence factor of *P. aeruginosa*. MG concentration-dependently suppressed the expression of *lasI*/*R*, *rhlI*/*R*, and *pqsA* of *P. aeruginosa* and was non-toxic in *in vitro* study. This is the first report of the anti-QS mechanism of MG.

## Introduction

Quorum sensing (QS) is an inter-cellular communication system of bacteria that is used to collectively control group behaviors^1, 2^. This process depends on the production, release, and group-wide detection of signal molecules which are known as autoinducers. The autoinducers in gram-negative bacteria are typically homoserine lactones (HSLs)^1, 2^. LuxI-type enzymes involved in the production of HSLs, and LuxR-type cytoplasmic proteins act as the receptors of HSL^1, 2^. LuxR-type receptors can be stabilized by binding with autoinducers, and this stabilization enables the dimerization, binding of DNA, and the transcription of QS target genes^3, 4^. LuxI/R signaling cascades are essential for the virulence in many pathogenic bacteria, and the virulence of these bacteria can be prevented by disabling these circuits with small molecules^2^.

*C. violaceum*is an aquatic, saprophyte, gram-negative bacterium that occasionally acts as a pathogen of extreme virulence, and causes fatal septicemia, lung and liver abscesses, and skin lesions^5^. *C. violaceum* produces violacein pigment in response to QS regulated gene expression^6^. Considering this characteristic, this bacterium is widely used to study the inhibition of acyl homoserine lactone (AHL)-dependent QS by diverse compounds^7–9^. The utilization of this bacterium is also very common in the assessment of short chain AHL production, because of the tight AHLs-QS control over the production of violacein pigment^10^. *Pseudomonas aeruginosa* is an opportunistic pathogen that causes morbidity and mortality in immune-compromised patients such as cystic fibrosis, AIDS, cancer patients and severe burn victims^11^. This organism depends on two key LuxI/R QS systems, namely Las and Rhl systems, for organizing simultaneous production of biofilm and virulence factors^12^. The autoinducer molecule, 3-oxo-C_12_-HSL is produced by LasI and responded by LasR in *P. aeruginosa*. The LasR:3-oxo-C_12_-HSL complex activates the transcription of many genes including *rhlR*, that encodes a second QS receptor^13^. RhlR binds to the autoinducer C_4_-HSL, the product of RhlI. RhlR:C_4_-HSL also directs a large regulon of genes, some of which are also members of the LasR regulon^13^. This tandem regulatory arrangement allows LasI/R to control the first stage of QS-controlled gene expression, and RhlI/R to control the second one. Because, LasR activates the expression of *rhlR*, and thus, the expression of both LasR- and RhlR-regulated target genes are reduced by the deletion of *lasR* ^14, 15^.

QS-dependent regulation of gene expression controls a wide variety of phenotypes including bioluminescence, biofilm formation, drug resistant, virulence factors expression, and motility. Therefore, the inhibition of QS is considered to be a new promising target of antimicrobial pathway as anti-virulence compounds, which can repress the gene expression and are essential for the basic metabolism *in vitro*, rather than the microorganism itself^16^. QS attracts attention as a promising anti-pathogenic drug target rather than antibacterial, notably preventing the emergence of drug resistant bacteria, because many pathogenic bacteria employ QS to regulate their pathogenicity and virulence factor production^17, 18^. Molecules that target QS have been proposed as an anti-virulence strategy. Efforts to identify small molecule inhibitors of QS were reviewed recently^11, 19–24^. Generally, the QS inhibitors work by the (i) inhibition of QS signal molecule biosynthesis, (ii) degradation and inactivation of QS signal molecules, and (iii) inhibition of signal molecule detection by receptors. Investigators have identified a variety of inhibitors by performing cell-based screens, or by synthesizing AHL analogs^22, 25–28^.

**Figure 1:**
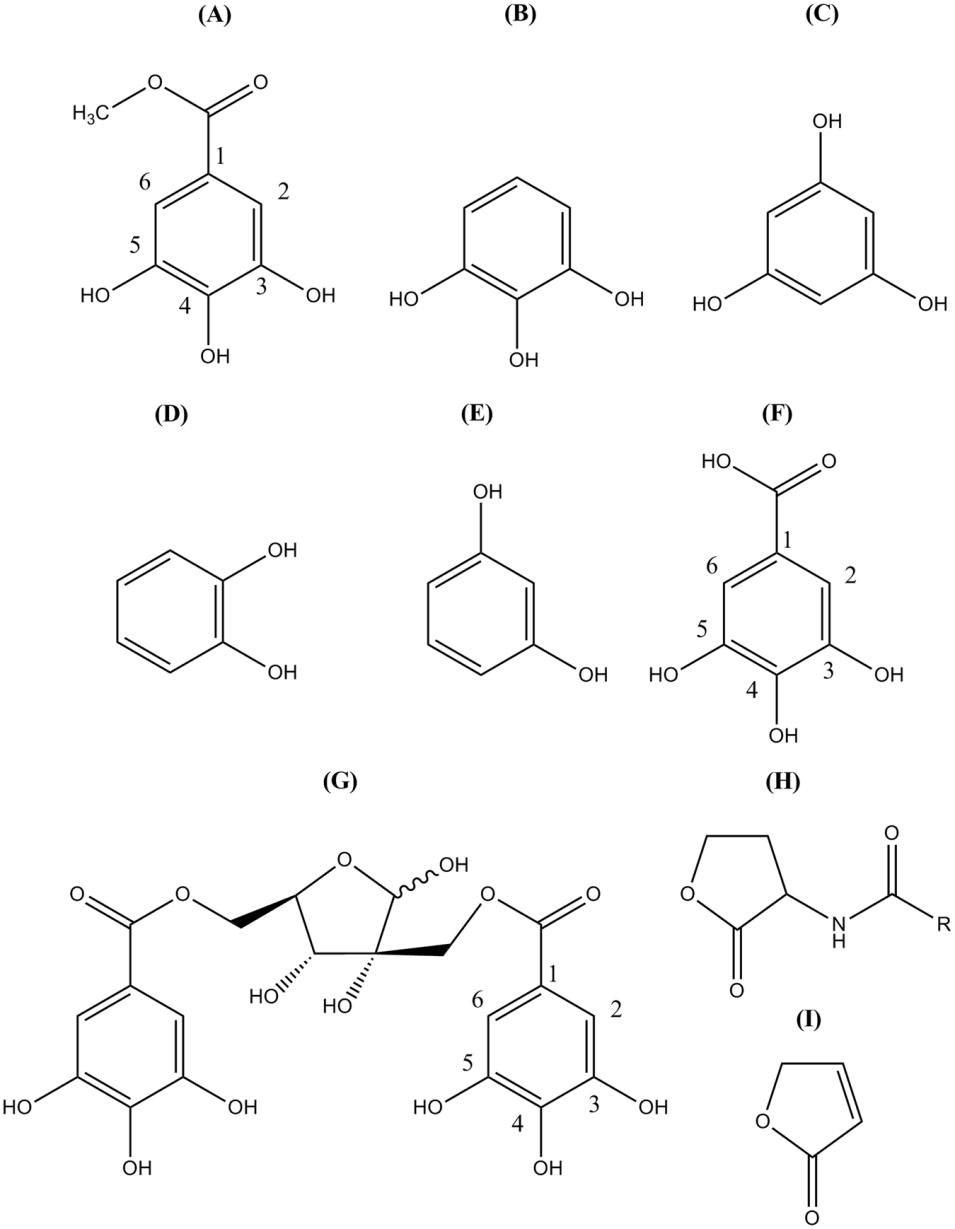
Chemical structures of selected phenolic compounds and their similarity/dissimilarity with autoinducer molecule. Chemical structure of (**A**) methyl gallate, (**B**) pyrogallol, (**C**) phloroglucinol, (**D**) pyrocatechol, **(E**) resorcinol, (**F**) gallic acid, (**G**) hamamelitannin, (**H**) acyl-homoserine lactone, and (**I**) furanone. Structures were drawn by ChemDraw software.

For this study, we have selected 5 phenolic compounds (MG, pyrogallol, pyrocatechol, resorcinol and phloroglucinol) (Fig. 1). These phenolic compounds commonly contain phenol ring with attached hydroxyl groups, but they differ in the number and positions of these additional hydroxyl groups. Moreover, the MG has a methyl ester group at its 1 position. On the other hand, the QS signal molecule, AHL has a lactone ring and amide group. So, these phenolic compounds are different than AHL considering their basic structures and their attached functional groups or side chain. The furanone also has lactone ring as like in AHL and is used as AHL analogue. But, this compound is different than the above mentioned phenolic compounds in view of their basic structure. The pyrogallol, a trihydroxybenzene having the same central structure like MG except of having methyl ester was reported for QS inhibition in *Vibrio harveyi*, where the QS is regulated by three pathways^29, 30^. Hamamelitannin, a polyphenolic compound with the basic galloyl moiety showed QS inhibitions against *S. aureus* ^31^.

Methyl Gallate is reported to have antioxidant, anticancer, antivirus, anti-inflammatory, antiasthmatic and vasodilative activities^32^. This compound exhibited substantial inhibitory effects on the protein synthesis and succinate dehydrogenase activity in a plant pathogen^33^. Treatment of *Ralstonia solanacearum* with MG showed that this compound can interfere with their respiration and metabolism^33^. The three hydroxyl groups at the phenyl ring of MG correspond to the important portion of the molecule for activity (pharmacophore)^30^. This information is supported by Ni *et al*. who evaluated pyrogallol derivatives, and showed that they confer QS antagonist activity^30^. The alkyl-ester of gallic acid was responsible to show its activities specifically against biofilm of *S. mutans*, which initiates this speculation that the methyl ester of gallic acid (MG) may also work against biofilm of *P. aeruginosa* (PAO1)^34^. Additionally, many phenols can non-specifically affect molecular targets of microorganisms. They contain a large number of hydroxyls, therefore can form protonic and ionic bonds and combine with many proteins of some bio-organisms like enzymes, carriers, ion channels and receptors, deactivating them and consequently exhibit bacterial inhibition^35^.

The existence of large proportion of MG and pyrogallol in ethyl acetate fraction of *Nymphaea tetragona* 50% methanol extract (NTME) has been determined by chromatographic analysis (Supplementary Fig. 1) in our earlier study together with the synergistic antibacterial and anti-QS effect of NTME^36^. Previously, we also evaluated the potentials of MG-containing NTME in the inhibition of QS as well as virulence factors in *C. violaceum* (ATCC12472) and *P. aeruginosa* (PAO1)^37^. At the initial stage of the current study, it was intended to develop precise QSIs by evaluating the QS inhibition potentials of 5 phenolic compounds (MG, pyrogallol, pyrocatechol, resorcinol and phloroglucinol), targeting mainly to the opportunistic compound MG. We also aimed to study the influence of the most potent QSI among these 5 phenolic compounds on *P. aeruginosa* QS-regulated virulence factors production, motility and biofilm formation. Furthermore, the molecular and genetic mechanisms underlying to the suspected effects of the most potent QS inhibitors were aimed to evaluate.

## Results

### MIC and MBC of phenolic compounds

The MIC and MBC values of structurally related 5 phenolic compounds against three strains of *C. violaceum* (ATCC12472, ATCC31532 and CV026), and *P. aeruginosa* (PAO1) are presented in Table 1. The MICs of MG, phloroglucinol, pyrocatechol, pyrogallol, and resorcinol were 32–512 μg/mL, 2048 μg/mL, 64–512 μg/mL, 4–64 μg/mL and 2048 μg/mL, respectively. The MBC values of all these compounds were 2- to 4-times higher than MIC values against these strains.

**Table 1:** Minimum inhibitory concentration (MIC) and minimum bacteriocidal concentration (MBC) of phenolic compounds against three strains (ATCC12472, ATCC31532, and CV026) of *Chromobacterium violaceum*, and *Pseudomonas aeruginosa* (PAO1).

Name of Drug	Concentrations	*Chromobacterium violaceum*			*Pseudomonas aeruginosa*
		ATCC12472	ATCC31532	CV026	PAO1
Methyl Gallate (μg/mL)	MIC	128	32	64	512
	MBC	256	64	128	2048
Phloroglucinol (μg/mL)	MIC	2048	2048	2048	2048
	MBC	8192	4096	4096	8192
Pyrocatechol (μg/mL)	MIC	64	128	64	256
	MBC	128	256	128	512
Pyrogallol (μg/mL)	MIC	8	4	4	64
	MBC	16	8	8	256
Resorcinol (μg/mL)	MIC	2048	1024	2048	2048
	MBC	4096	2048	4096	4096
Norfloxacin (μg/mL)	MIC	0.016	0.016	0.016	0.25
	MBC	0.0625	0.032	0.0625	1

### Quorum sensing inhibition of phenolic compounds

**Figure 2:**
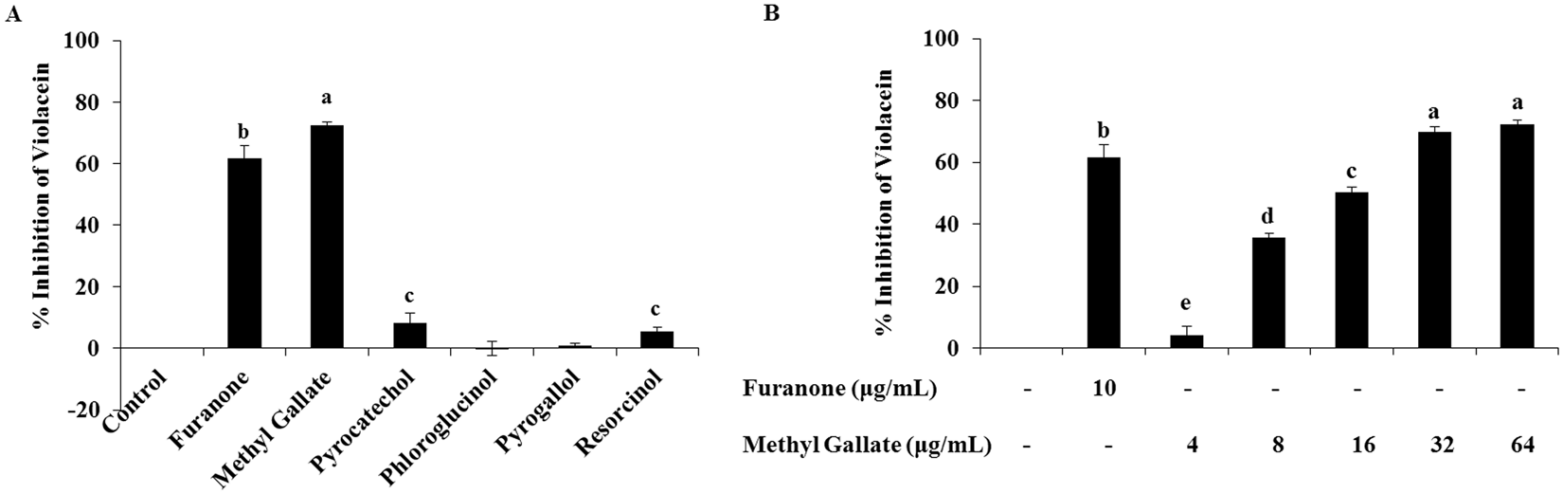
Quantitative quorum sensing inhibition ensuing percent (%) violacein inhibition of (**A**) 5 phenolic compounds (methyl gallate, phloroglucinol, pyrocatechol, pyrogallol, and resorcinol), and (**B**) Concentration-dependent percent (%) violacein inhibition of methyl gallate. Data represent the mean ± SD of triple determinations. Different superscript characters indicate significant differences at *P* < 0.05.

The inhibition diameters of violacein pigment of *C. violaceum* in presence of phenolic compounds are presented in Table 2. MG significantly inhibited the production of violacein pigment, even with the lowest concentration (15 μg/disk) used in this assay (Supplementary Fig. 2). Pyrogallol in all tested concentrations showed bactericidal effect (clear zone). Pyrocatechol showed a small diameter of pigment inhibition zone with 60 μg/disk. Other compounds with the tested concentrations were not found to be effective enough to interfere in the production of violacein by *C. violaceum*. Furanone confirmed the violacein pigment inhibition, whereas the negative control and tetracycline displayed no effect and bactericidal effect, respectively. In quantitative determination, MG, pyrocatechol and resorcinol were found to inhibit the production of violacein about 72.24, 8.39 and 5.49% respectively, compared to the drug-free control (Fig. 2A). Phloroglucinol and pyrogallol could not inhibit violacein production significantly. The supplementation of MG in *C. violaceum* culture showed concentration dependent effect on violacein inhibition (Fig. 2B).

**Table 2:** Quorum sensing inhibition activity (as pigment inhibition zone diameters) of phenolic compounds against *Chromobacterium violaceum* ATCC12472.

Drug	Concentrations	Pigment Inhibition Diameter (mm)
	(µg/disk)	Mean ± SD
Tetracycline	10	20.33 ± 1.53*
Furanone	10	14.00 ± 1.00^c^
Normal Saline	—	—
Methyl gallate	15	12.67 ± 1.15^c^
	30	16.67 ± 1.15^b^
	60	23.33 ± 2.08^a^
Phloroglucinol	15	0.00 ± 0.00
	30	0.00 ± 0.00
	60	0.00 ± 0.00
Pyrocatechol	15	0.00 ± 0.00
	30	0.00 ± 0.00
	60	9.33 ± 0.58^d^
Pyrogallol	15	10.33 ± 0.58*
	30	11.00 ± 1.00*
	60	13.33 ± 0.58*
Resorcinol	15	0.00 ± 0.00
	30	0.00 ± 0.00
	60	0.00 ± 0.00

Different superscript alphabets indicate significant difference (*P* > 0.05) in violacein pigment inhibition diameter. *Diameter of clear zone. Data shown represent the mean ± SD of three replicates.

### Effects of MG on AHL synthesis and AHL activity

The effects of MG on the synthesis of AHL and on the activity of AHL were determined passively from the intensity/concentration of violacein pigment, which is the general measure of quorum sensing that can be happened if AHLs bind to their cognate receptors^6^. Two strains of *C. violaceum* (ATCC31532 and CV026) were used in this study. *C. violaceum* ATCC31532 is a C6-HSL over-producer strain, which can only synthesis the signal molecule but, cannot respond to that signal molecule^38, 39^. Conversely, *C. violaceum* biosensor strain (CV026) is a mini-Tn5 mutant of *C. violaceum* ATCC31532, which does not produce signal molecule such as C6-HSL, but has CviR receptor and shows response to the exogenous C6-HSL^38, 39^. In this assay, the presence of violate violacein color represents the availability of AHL and the binding of the AHL molecules to the AHL receptor, whereas the absence of violate color indicates the lack of AHL and/or AHL-receptor binding^40^.

It was found in the qualitative agar diffusion assay that MG at sub-lethal concentrations reduced AHL production from the AHL over-producer strain (Supplementary Fig. 3A) as indicated by the absence or indistinct pigmentation of violacein (Supplementary Fig. 4) produced by the biosensor strain (CV026). MG also appeared to interfere with the activity of AHL in the CV026/31532 bioassay system which was assessed by the production of low levels of AHL-mediated violacein pigment in CV026 AHL biosensor (Supplementary Fig. 3B). Our results indicated that compared to the control which did not contain any drug, the AHL-mediated violacein pigment production in CV026 and its activity were much lower in the presence of MG (Supplementary Fig. 3).

**Figure 3:**
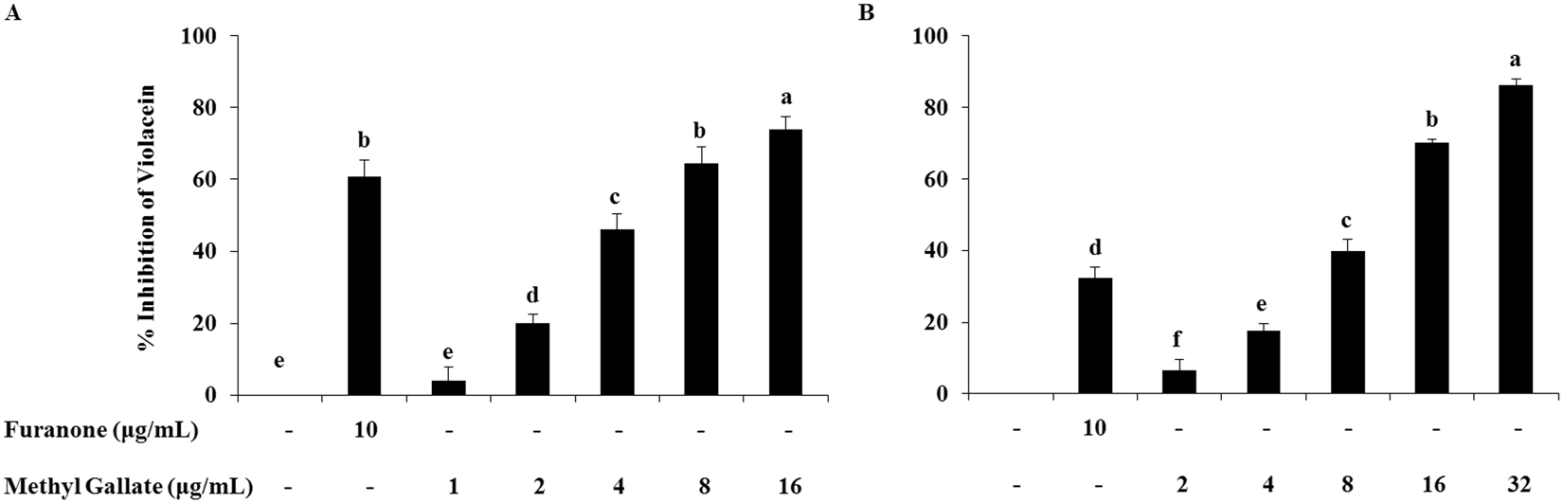
A quantitative determination of the effect of methyl gallate on AHL synthesis and their activity. Effects on (**A**) AHL synthesis, and (**B**) AHL activity. Data represent the mean ± SD of triple experiments. Different superscript letters indicate significant differences at *P* < 0.05.

Together with the qualitative determination, we quantified the extent of MG to both in the inhibition of AHL synthesis by the AHL over producer strain ATCC31532, and in the interference with the AHL activity in biosensor strain (CV026) by utilizing two independent assays with the CV026/31532 bioassay in broth media. AHLs were extracted from *C. violaceum* ATCC31532 after treating them with different sub-MIC concentrations of MG and incubated the biosensor strain CV026 in presence of those extracted AHLs without any drug compounds to determine the effects of MG on the synthesis of AHL. The synthesis of AHL were inhibited about 20%, 46%, 64%, and 74% in contrast to the drug-free control, by the supplementation of 2, 4, 8 and 16 μg/mL of MG, respectively (Fig. 3A). In determining the effects of MG on the activity of AHL, we incubated the AHL over producer strain *C. violaceum* ATCC31532 without any treatment and extracted the AHL. The biosensor strain CV026 were incubated with those extracted AHL and treated at the same time with different sub-MIC of MG. The activity of AHL was restricted about 7%, 18%, 40%, 70%, and 86% compared to the drug-free control, in presence of 2, 4, 8, 16 and 32 μg/mL of MG, correspondingly (Fig. 3B). The extents of synthesized AHL and their activity were determined by the OD values of violacein. The result indicated that the MG more effectively inhibited the activity of AHL, compared to the inhibition of AHL synthesis.

### Effects of phenolic compounds on swarming motility

**Figure 4:**
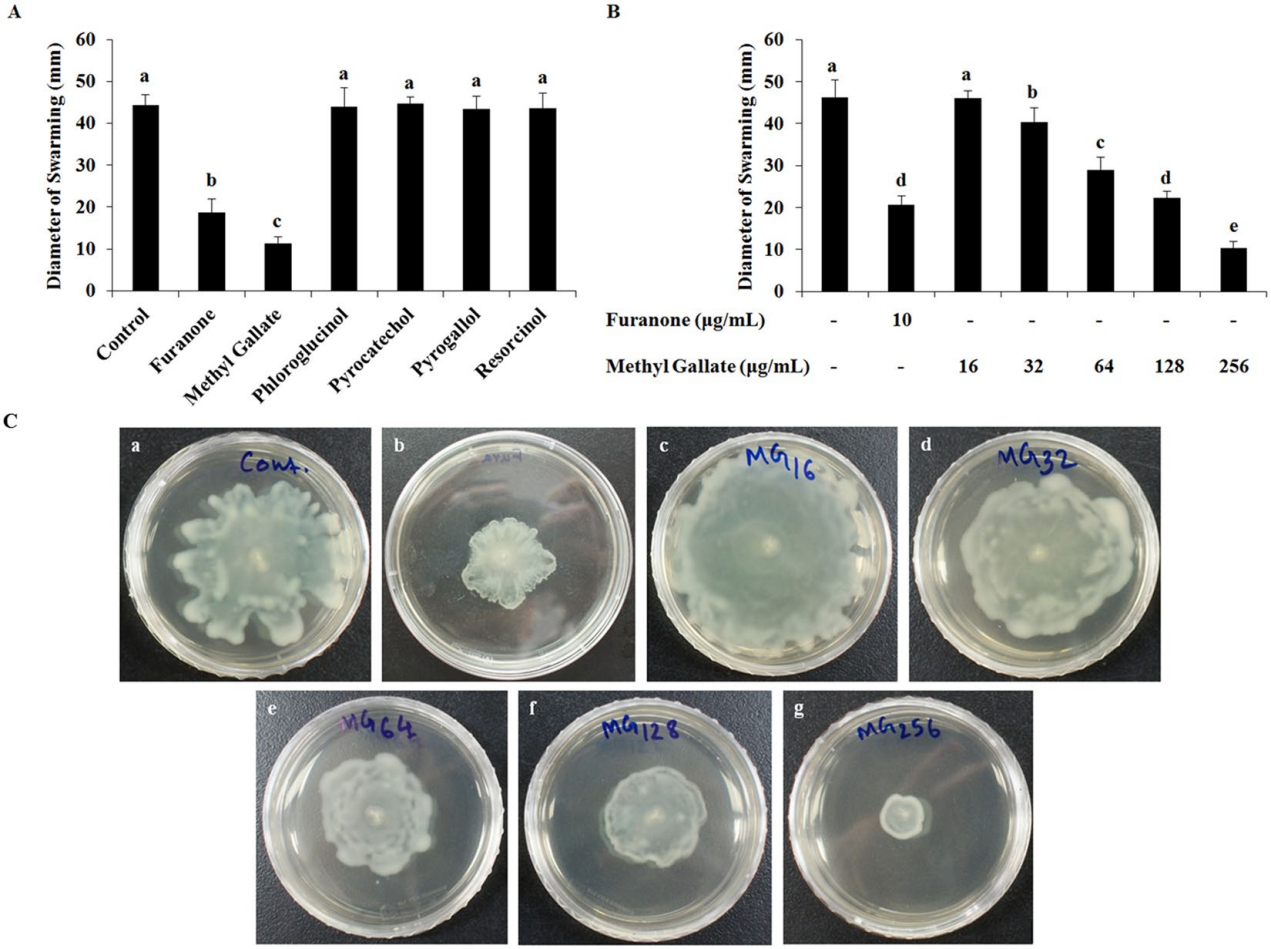
Swarming motility of *P. aeruginosa* (PAO1) in presence of five phenolic compounds. Swarming zone diameters of *P. aeruginosa* (PAO1) in presence of (**A**) 1/2 × MIC of methyl gallate, phloroglucinol, pyrocatechol, pyrogallol and resorcinol, (**B**) 16~256 µg/mL of methyl gallate. (**C**) Representative swarm plates incubated with the supplementation of increasing concentrations (16~256 µg/mL) of methyl gallate. Data represent the mean ± SD of triple determinations. Different superscript letters are significantly different at *P* < 0.05.

The effect of these phenolic compounds in the inhibition of swarming motility of *P. aeruginosa* (PAO1) was examined, and the results showed that only MG among these 5 compounds significantly restricted the swarming motility in *P. aeruginosa* (Fig. 4A). Moreover, MG inhibited the swarming motility of *P. aeruginosa* in a dose-dependent manner at sub-MIC (Fig. 4B,C). The diameter of swarm zones were about 46, 40, 29, 22, and 10 mm, respectively in presence of 16, 32, 64, 128, and 256 μg/mL of MG, whereas the swarm zone diameter in non-treated sample was about 46 mm (Fig. 4B).

### Effects of MG on biofilm formation and its viability

**Figure 5:**
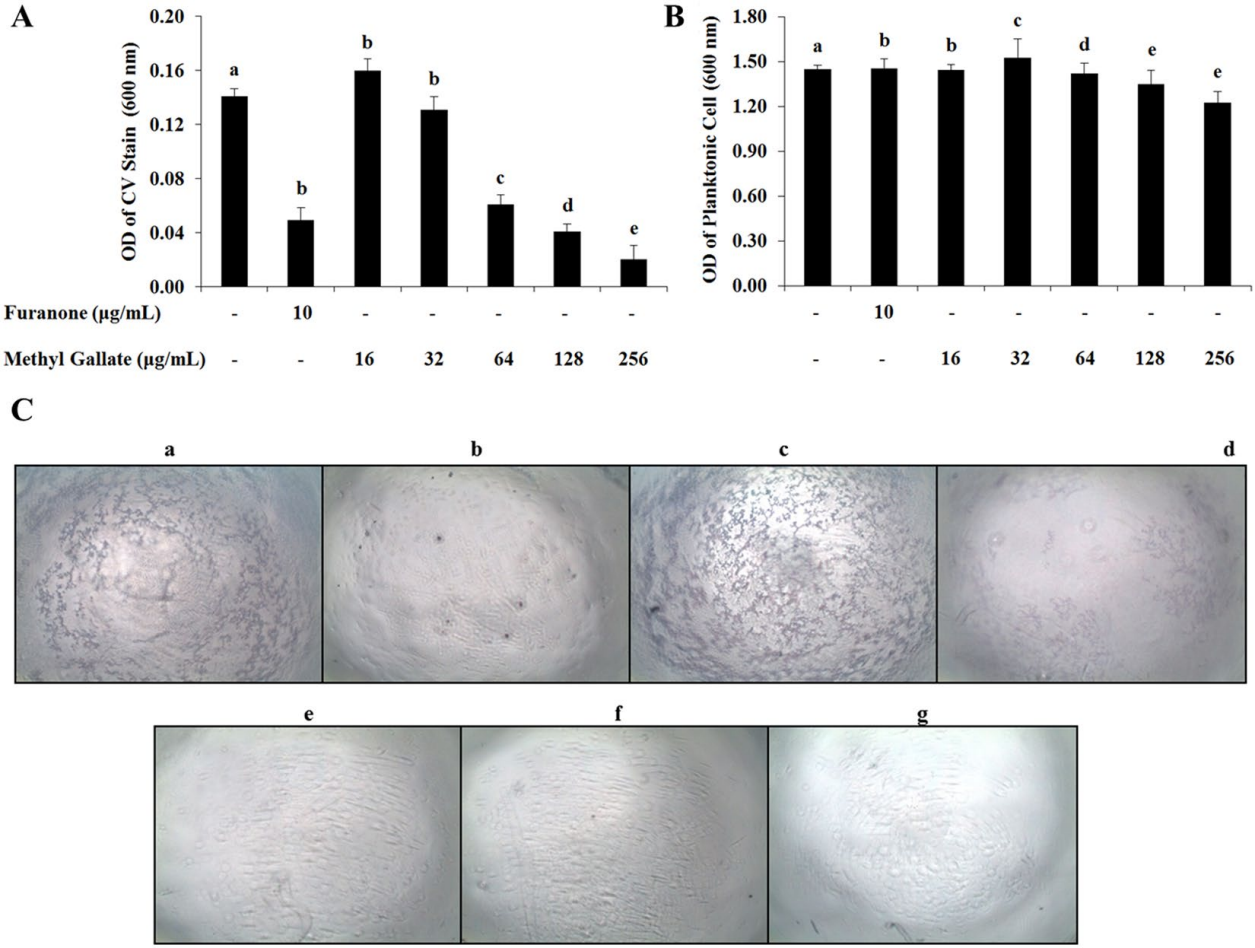
Effects of methyl gallate in the inhibition of biofilm formation. The OD values of (**A**) crystal violet solution extracted from the biofilm cell surface, and (**B**) planktonic cell suspension at a wavelength of 600 nm. (**C**) Representative photographs of crystal violet stained biofilm cells on 96-well surface under light microscope where, (a) control; (b) furanone 10 µg/mL; (c–g) 16~256 µg/mL of methyl gallate. Violet spots on surface indicate biofilm. Data represent the mean ± SD of triplicate assays. Different superscript letters indicate significant differences at *P* < 0.05.

The impact of MG on biofilm formation was determined by keeping the bacterial culture in static condition with/without MG to develop biofilms. The result showed that the biofilm formation was inhibited concentration-dependently with the addition of sub-MIC of MG (Fig. 5A). The optical density (OD) values of crystal violet stain that retained on biofilms were about 0.160, 0.131, 0.060, 0.041 and 0.020 with the treatment of 16, 32, 64, 128, and 256 μg/mL of MG, respectively. The photographs of crystal violet stained-biofilms on the surface of wells also showed that the MG concentration-dependently inhibited the formation of biofilm (Fig. 5C). The effect of MG on planktonic cell growth was also determined (Fig. 5B). There was no significant variation in OD values of planktonic cell suspension indicates that the compound has no impact on planktonic cell growth.

**Figure 6:**
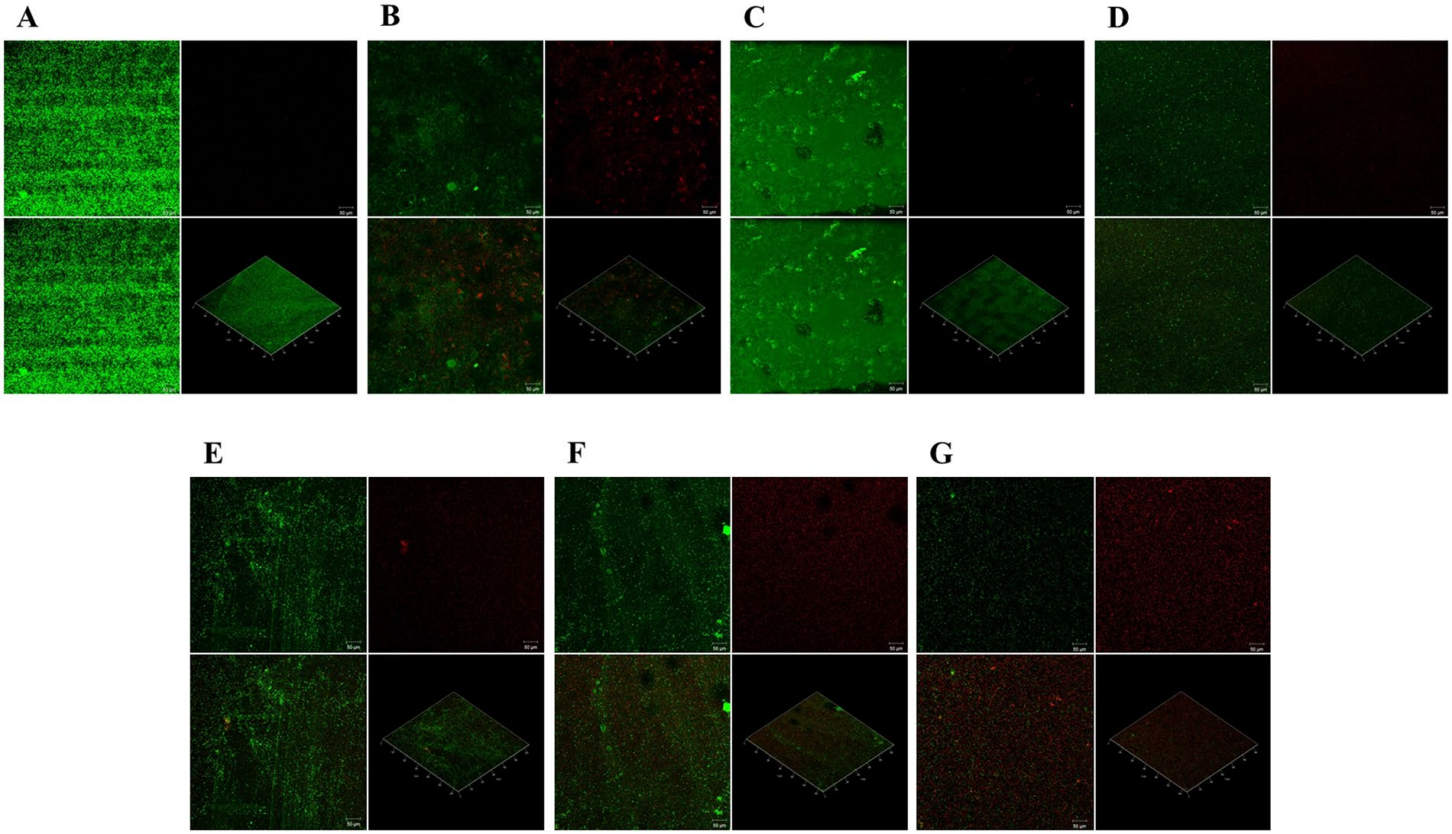
Viability of biofilm in presence of 16~256 µg/mL of methyl gallate. The confocal microscopic images of LIVE/DEAD stained biofilm in (**A**) drug free control; biofilm treated with (**B**) furanone 10 µg/mL; (**C**) 16 µg/mL, (**D**) 32 µg/mL, (**E**) 64 µg/mL, (**F**) 128 µg/mL, and (**G**) 256 µg/mL of methyl gallate. The viability of the biofilms was assessed using BacLight LIVE/DEAD stain (green: live cells, red: dead cells). In each image, the segment at below right side shows three dimensional and other three segments shows two dimensional images.

The viability of biofilm in presence of MG was determined by staining the biofilm with BacLight live/dead stain and by scanning in confocal microscope. The confocal micrograph of 48 h biofilm of *P. aeruginosa* treated with MG for 24 h displayed a lower proportion of live cells (cells that stained green) than that observed in the untreated control (Fig. 6). It is clearly visible that almost all cells were alive in the control biofilm. About 0.00%, 4.00%, 12.00%, 45.00%, and 70.00% dead cells (cells that stained red) were evident from the treatment with 16, 32, 64, 128, and 256 μg/mL of MG, respectively.

### Effects of MG on the Inhibition of virulence factor production

**Figure 7:**
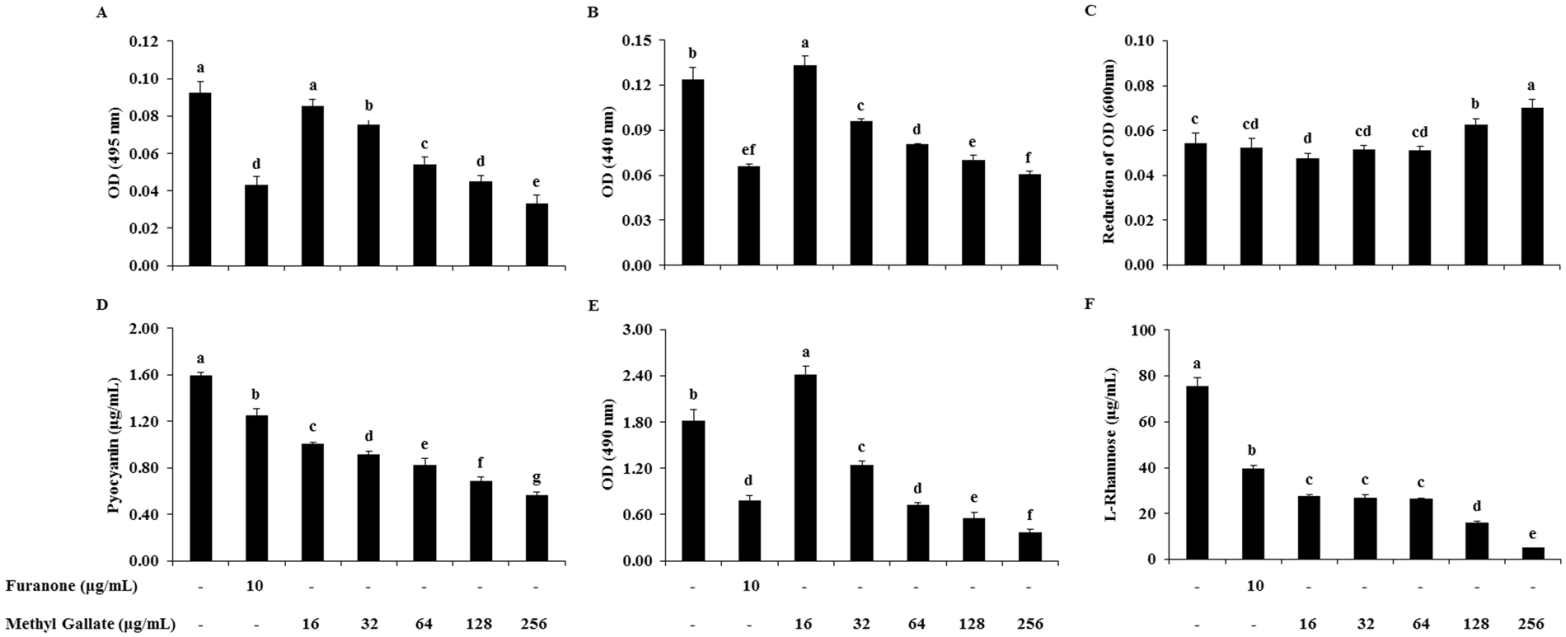
Effects of methyl gallate on virulence factors production. (**A**) Elastolytic activity, (**B**) Total proteolytic activity, (**C**) LasA Staphylolytic activity, (**D**) Pyocyanin production, (**E**) Exopolysaccharide production, (**F**) L-rhamnose production in presence of increasing concentrations (16~256 µg/mL) of methyl gallate. Data represent the mean ± SD of triplicate analyses. Different superscript letters indicate significant differences at *P* < 0.05.

The potentials of MG in down-regulating QS-associated virulence factors of *P. aeruginosa* were measured and are shown in Fig. 7. The activity of elastase, total protease, pyocyanin, exopolysaccharide, and rhamnose in *P. aeruginosa* (PAO1) were inhibited by MG with a dose-dependent manner. The supplementation of MG (16~256 μg/mL) in *P. aeruginosa* (PAO1) culture inhibited the activity of elastase (8~64)%, total protease (−7~51)%, pyocyanin (37~64)%, exopolysaccharide (−33~79)%, and rhamnose (63~93)%, compared to the activity found in drug-free control. The sub-MICs of MG above than 1/32 × MIC (16 μg/mL), concentration-dependently restricted the activities of these 5 virulent factors, and 1/32 × MIC of MG in *P. aeruginosa* increased the activity of total protease and exopolysaccharide compared to the non-treated control. On the other hand, LasA Staphylolytic activity was not inhibited in sub-MICs of MG (Fig. 7).

### Effects of MG on QS gene expression

**Figure 8:**
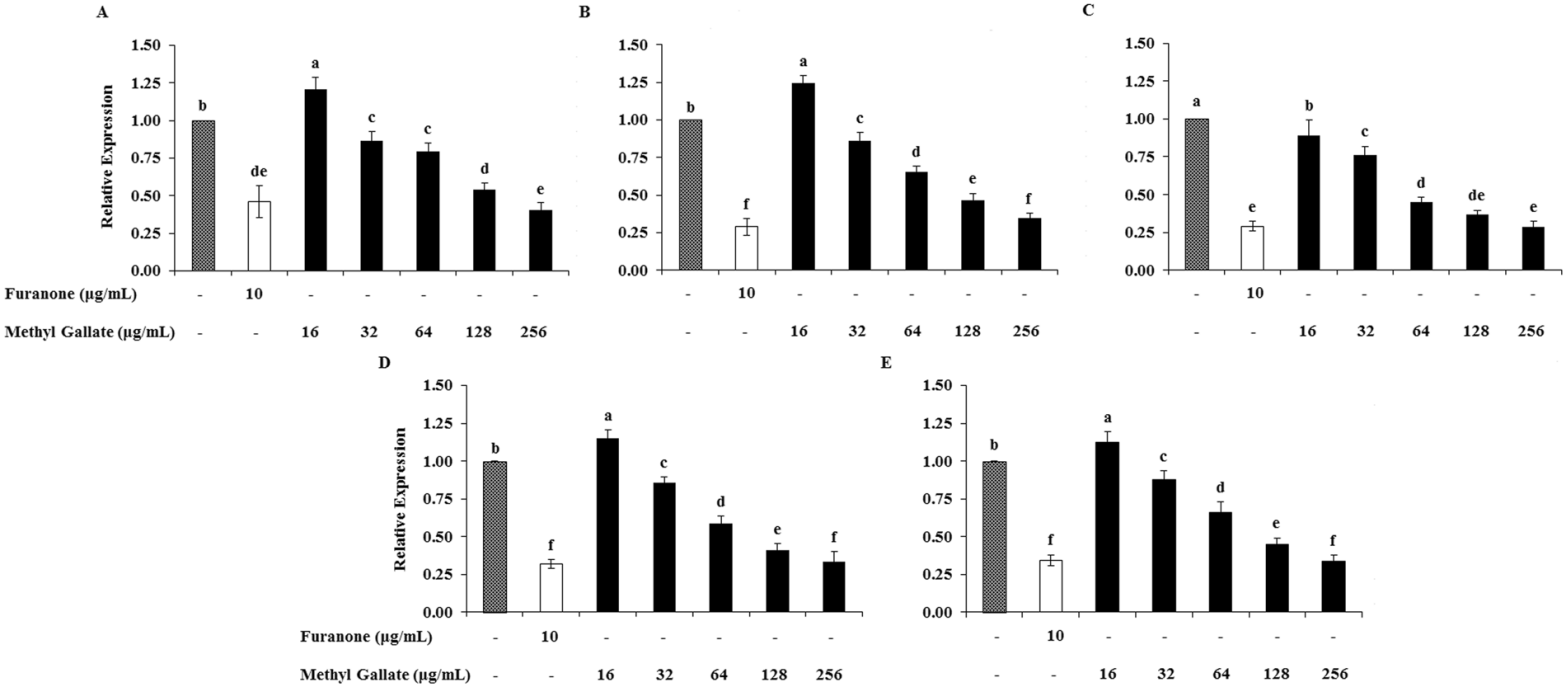
Relative expressions of quorum sensing regulated genes of *P. aeruginosa* (PAO1) in presence of increasing concentrations (16~256 µg/mL) of methyl gallate. (**A**) *lasI*, (**B**) *lasR*, (**C**) *rhlI*, (**D**) *rhlR*, and (**E**) *pqsA*. Data represent the mean ± SD of triplicate analyses. Different superscript letters indicate significant differences at *P* < 0.05.

In *P. aeruginosa* (PAO1), four regulatory genes (*lasI*, *lasR*, *rhlI*, *rhlR*) are very crucial to control the expression of many factors such as the virulence, pathogenicity, and biofilm development. The expressions of many other regulatory genes involve in QS are also controlled by these four particular genes^41^. Another important regulatory gene *pqs*A which is regulated by the *las* system and also regulates the *rhl* system^42, 43^. In addition to these regulatory genes, other QS regulator genes have been identified. But, their roles in QS are not fully understood yet^44, 45^. Thus, we evaluated the expressions of these 5 genes in this study. The expressions of QS-regulatory genes (*lasI*, *lasR*, *rhlI*, *rhlR* and *pqsA*) of *P. aeruginosa* (PAO1) were down regulated concentration-dependently and significantly when incubated this bacterium in presence of MG. Real time PCR revealed that 59.85 ± 0.60, 65.41 ± 0.65, 71.58 ± 0.72, 66.68 ± 0.67, and 66.00 ± 0.66% expressions of *lasI*, *lasR*, *rhlI*, *rhlR* and *pqsA*, respectively reduced with the treatment of 1/2 × MIC (256 μg/mL) of MG compared to the expressions recorded in drug-free control (Fig. 8). Two fold reduced expressions of all these genes except for *lasI* were found in 1/4 × MIC (128 μg/mL) of MG-supplemented culture compared to drug-free control. The optimization of incubation time for better expression of *lasR* genes in *P. aeruginosa* (PAO1), and the optimization of the amount of 3-oxo-C_12_-HSL need to supplement for better expression of QS genes are presented in Supplementary Fig. 5.

### Effects of MG on cell viability

**Figure 9:**
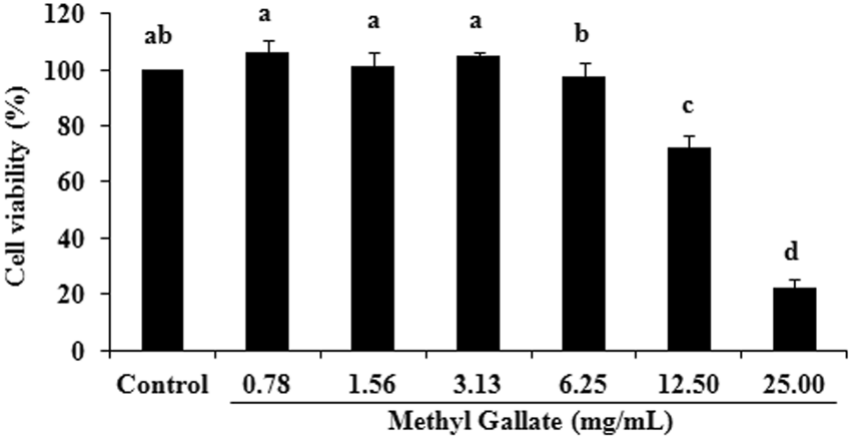
Effect of methyl gallate on the viability of Raw 264.7 cell. Data represent the mean ± SD of triple assays. Different superscript letters indicate significant differences at *P* < 0.05.

The effect of MG on the viability of RAW 264.7 cell line was evaluated to determine the safety profile of this compound. The effects of different concentrations (0.78–25.00 mg/mL) of MG on RAW 264.7 cell line are presented in Fig. 9. The MG < 6.25 mg/mL did not produce significant toxic effect. Methyl gallate concentration-dependently suppressed the viability of RAW 264.7 cells, when exposed in 6.25–25.00 mg/mL for 24 h. The reductions of viable cells in presence of 6.25, 12.50 and 25.00 mg/mL of MG were 2.34%, 27.78%, and 77.66% of the non-treated control cells, respectively. The IC_50_ value of MG in RAW 264.7 cells was 16.94 mg/mL.

## Discussion

Antimicrobials, whether bactericidal or growth inhibitory, place strong selective pressures on bacteria to develop resistance, and their widespread use has accelerated the emergence of resistant pathogens. Though there is a desperate need for the discovery and production of novel antimicrobials, an investment in developing new strategies that target mechanisms of virulence rather than bacterial growth may offer therapeutic potential that is more sustainable. These so-called anti-infective therapies would ideally target bacterial pathways that lead to disease but not interfere with bacterial growth. Thus, this study explored the interference of 5 phenolic compounds with AHL-mediated QS and demonstrated that MG among these compounds suppresses the regulation of QS through the inhibition of both AHL synthesis and the activity of synthesized AHL in *C. violaceum*. This compound was also found to repress the expression of genes encoding the virulence factors elastase, protease, exopolysaccharide and rhamnose, and prevents swarming motility and biofilm formation in *P. aeruginosa* (PAO1). We have employed comprehensive biochemical, molecular and genetic techniques to support our conclusions.

All the phenolic compounds evaluated in this study exhibited different degrees of bacterial inhibition against *C. violaceum* and *P. aeruginosa* (Table 1). A general overview of the MIC results showed that the tested phenolic compounds were comparatively less effective than a classical antimicrobial (e.g. norfloxacin) against these Gram-negative bacteria. Pyrogallol was found to be the most effective antibacterial agent, followed by pyrocatechol, MG, resorcinol, and phloroglucinol. The MBC values of these drugs are 2–4 folds higher than the MIC values against these bacteria which indicate the rational antibacterial therapeutic windows of these drugs.

The QS inhibition potentials of these phenolic compounds against *C. violaceum* were determined by disk agar diffusion and broth incubation assays, which demonstrate the promising anti-QS activity of MG. In the disk diffusion assay, the clear zone without any bacterial cells around the disk represents antibacterial activity, which is caused by tetracycline in this study (Supplementary Fig. 2). Furanone 10 μg/disk, different concentrations (15, 30 and 60 μg/disk) of MG, and 60 μg/disk of pyrocatechol showed QS inhibition as they only inhibited the violet violacein pigment around the disk without affecting the bacterial cell (Table 2). The QS inhibition effect of MG presented in Supplementary Fig. 2 and Table 2 is comparable with the previously reported anti-QS activity of caffeine and mycofabricated biosilver nanoparticles^46–48^. In the quantitative study, violacein pigment production of *C. violaceum* was also inhibited significantly and concentration-dependently by MG (Fig. 2) whereas the other phenolic compounds did not noticeably inhibit the violacein pigment production. Interestingly, the sub-MIC of MG repressed the violet violacein production of *C. violaceum* without restricting the growth of bacterial cells. Natural and synthetic molecules were also reported to inhibit violacein production concentration-dependently without inhibiting bacterial growth^49–51^, which justifies the potentials of MG as QSI (Fig. 2).

The results of the qualitative and quantitative assays in the present study (Fig. 3, and Supplementary Fig. 3) have demonstrated that MG not only interferes with AHL activity, but also affects the synthesis of AHL. Effects of MG on the synthesis of AHL were determined by the OD value of violacein produced by the biosensor strain *C. violaceum* CV026 in presence of the AHLs that were extracted from the MG-treated cultures of *C. violaceum* ATCC31532. Conversely, the OD value of violacein produced in *C. violaceum* CV026 culture treated with sub-MICs of MG with added AHL from untreated cultures of *C. violaceum* ATCC31532 were measured to determine the effect of MG on the activity of AHL. The effect of MG on the activity of AHL is independent of the effects on AHL synthesis (as explained in result section). Hence, the results of this assay indicate that MG can inhibit QS either by accelerating the degradation or inactivation of AHL molecule, or reducing the ability of bacteria to synthesize AHL molecules or by inhibiting the AHL-receptor binding^52–54^. The suppression of AHL synthesis can happen by decreasing the expression of the LaxI/LasI family of synthases. While these mechanisms are most likely, it is also possible that MG may act as ligand or may directly restrict the virulence factor production in bacteria in a QS-independent manner.

It is observed in different studies that the compounds which inhibit QS in the *Chromobacterium* system also inhibit the production and/or secretion of QS-related virulence factors in *Pseudomonas* ^55^. The results of this study also suggest that MG among these phenolic compounds inhibited QS, as well as decreased the production of QS-regulated virulence factors in *P. aeruginosa*. In addition to flagella and pili, swarming of *P. aeruginosa* also requires the production of two bio-surfactants; rhamnolipids and 3-hydroxyalkonics acids^56^. It is reported in different studies that the production of rhamnolipids in *P. aeruginosa* (PAO1) is controlled by QS^57^. Our results (Fig. 4) indicate that only MG among these compounds showed observable inhibition against the swarming of *P. aeruginosa* with the formation of bacterial colony of short and undefined tendrils. Furthermore, the dose-dependent inhibition of swarming motility in the presence of MG suggests that the compound might has some effects on flagella-related processes, namely, flagella biosynthesis, rotation, and chemotaxis, which may lead to decrease in swarming activity.

*P*. *aeruginosa* has been shown to form organized, surface-attached microbial communities, called biofilms. This trait has been linked to pathogenicity of the organism in relation to pulmonary infections in cystic fibrosis. Several studies have previously demonstrated that swarming motility is required for biofilm dispersion and it is assumed from the results of swarming inhibition of MG that this compound might inhibit the dispersion of biofilm^58^. The staining of biofilm cells with crystal violate proved a concentration-dependent inhibition effect of MG on the formation and growth of biofilm (Fig. 5). This finding demonstrated that the compound in sub-MIC can inhibit the development of biofilm without affecting the planktonic cell growth. The surviving and dead biofilm populations were verified by live/dead staining, where the bacterial biofilms stained green are considered as live biofilm cell and those stained red are recognized as dead cells. The results clearly showed that MG can affect the viability of biofilm of *P. aeruginosa* in a concentration-dependent manner (Fig. 6). Biofilms are encased in a matrix composed of exopolysaccharide^59^.

There are three exopolysaccharides (alginate, Pel and Psl) produced by *P. aeruginosa*, and all of these exopolysaccharides have been found to contribute in biofilm formation^60^. Exopolysaccharide production in *P. aeruginosa* was significantly reduced with the treatment of MG (Fig. 7E). Earlier reports indicate the importance of pyocyanin in *P. aeruginosa* virulence^61^. Pyocyanin is a blue colored pigment produced by *P. aeruginosa*, which found in the sputum of patients suffering from cystic fibrosis, and has a great role in pulmonary tissue damage^62^. MG significantly reduced pyocyanin pigment production in *P. aeruginosa* (Fig. 7D). *P. aeruginosa* strains that overproduce pyocyanin are also known to produce other extracellular virulence factors, such as proteases, which are believed to play a major role in pathogenesis, especially during acute infections^63^. We determined that the incubation of *P. aeruginosa* in presence of MG also affects the production of two proteases, the LasB elastase and the LasA staphylolytic protease (Fig. 7A–C). *P. aeruginosa* elastase is a strong exotoxin that induces tissue damage; therefore, significant efforts are giving to develop chemical compounds that suppress elastase activity. LasA protease has a vital function in the pathogenesis of bacteria by means of host tissue degradation^37^.

Rhamnolipids have been detected in sputum from cystic fibrosis patients and are known to lyse polymorpho nuclear leukocytes (PMNs) and monocyte-derived macrophages, resulting in necrotic cell death^64^. They have role in the maintenance of mushroom-shaped macrocolonies and in the opening of channels in mature biofilm. Rhamnolipids also play a role in overcoming surface tension and allow swarming motility of *P. aeruginosa* ^65^. MG reduced rhamnolipid production significantly and concentration-dependently (Fig. 7F). Pyocyanin, elastase, protease and rhamnolipids are regarded as indicators of the optimal operation of QS regulon in *P. aeruginosa*. Reduction in their production level indicates the anti QS potential of MG. Both swarming and pyocyanin are produced and regulated by the *rhl* system in *P. aeruginosa*. Therefore, collectively inhibition of *P. aeruginosa* swarming motility and pyocyanin production suggests the presence of *rhl* inhibitor^61^. Total protease and elastase are directly controlled by the *las* system, and pyocyanin and rhamnolipid are mainly regulated by the *rhl* system^66^. The reduction in virulence factors controlled by 3-oxo-C_12_-HSL (elastase and protease) and C_4_HSL (rhamnolipids and pyocyanin) in *P. aeruginosa* (PAO1) indicates the reduction in both AHLs^67^.

The expression of *lasR* was comparatively better when they incubated for 16 h in presence of 100 μM of 3-oxo-C_12_-HSL (autoinducer). The incubation time was optimized for having the maximum expressions of QS-regulated genes, as the incubation times reported in different articles are different^68, 69^. The amount of autoinducer (3-oxo-C_12_-HSL) required to supplement was also optimized for the induction of *lasI*/*R* and *rhlI*/*R* cascade in *P. aeruginosa* (PAO1). Real-time polymerase chain reaction showed that MG concentration-dependently down regulated the transcriptions of autoinducer synthase (*lasI* and *rhlI*) and their cognate receptor (*lasR* and *rhlR*) genes, which resulted in attenuation of QS-regulated virulence activities, such as biofilm formation, and secretion of pyocyanin, elastase, protease, exopolysaccharides and rhamnolipid. The protease and elastase are *las*-controlled virulence factors, pyocyanin and rhamnolipid is controlled by *rhl* system^70, 71^. The reduction rates of elastolytic activity (64%), proteolytic activity (51%), pyocyanin (64%), exopolysaccharide (79%), and rhamnose (93%) for 1/2 × MIC of MG showed that this compound has stronger effect both on *lasR* and *rhlR*. Further, the *las* system contributes greatly to biofilm formation, and substantial down-regulated transcription of *lasR* and *lasI* may be directly responsible for the reduction of *P. aeruginosa* (PAO1) biofilms^72, 73^. More potent antagonists do not exhibit superior function in impeding virulence. Since, *lasR* and *rhlR* reciprocally control crucial virulence factors. Appropriately tuning rather than completely inhibiting their activities appear to hold the key to blocking pathogenesis. These results suggest that MG may possess *lasR* inhibitory activity against the virulence of *P. aeruginosa* (PAO1).

The evaluation of safety/toxicity profiles of any drug is desirable and essential with the investigation of pharmacological effects. Moreover, *P. aeruginosa* is especially dangerous in cystic fibrosis, and it is reported recently that the dysfunction of macrophage cell is considered to be the first step in cascade of events leading to chronic inflammation/infection in cystic fibrosis^74^. Alternatively, maintaining the macrophage cells in safe condition is essential to remain active the body’s own defensive system. Moreover, RAW 264.7 macrophage cell line is extensively used as a reliable cell model for inflammation research and for the study of macrophage cellular physiology because of their ease of culture, rapid growth rate and phenotypic resemblance to primary macrophages^75, 76^. Hence, the cytotoxic effect of MG was evaluated in RAW 264.7 macrophage cell line to determine whether this compound has any interference in the defensive mechanism or not. The *in vitro* cytotoxicity of the compound was evaluated using the MTT assay in the current study. The MG exhibited non-toxic effect against RAW 264.7 cells. The compound in 0.78–6.25 mg/mL did not interfere the viability of cells, and high concentrations (12.50 and 25.00 mg/mL) of this compound showed a certain level of reduction in the cell viability. The IC_50_ value of MG is 16.94 mg/mL. The effects of MG in cell viability were evaluated previously with different cell lines and found to have cytoprotective effect or no adverse effects in cell viability^77, 78^.

Among these phenolic compounds, only MG showed QS inhibition. There are many mechanisms by which AHLs can be inactivated^79^. It was found in previous studies that changing in the length of the acyl chain of AHL and/or substitution of the 3-oxo group with hydroxyl or methylene reduced the autoinducing activity^80^. Addition of a phenyl group to the acyl chain terminus resulted in compounds with antagonist activity^80^. Moreover, the substitutions to the HSL resulted in loss of activity presumably due to loss of autoinducer-receptor binding^80^. One or more than one of the above mentioned mechanisms may employ in the case of QS inhibition of MG. The result in this study predicts that the galloyl moiety may be the basic structure for QS inhibition and the attachment of methyl ester (COOCH_3_) in carbon 1 position possibly essential to exert the effect. This speculation is also supported by Pimenta *et al*.^34^. The galloyl moiety without methyl ester in that position may not produce QS inhibition as there was no QS inhibition by gallic acid (Fig. 1F) in another study^57^. It is predicted that the replacement of hydrogen ion from the carboxyl group of gallic acid with other functional groups and/or side chain may initiates the quorum sensing inhibitions against *P. aeruginosa* which were observed in epigallocatechin gallate, ellagic acid and tannic acid^81^. We hypothesized that derivatives of MG may also have QS inhibition potential as some derivatives are reported for biofilm inhibition in another study^34^.

This is the first report on the anti-QS properties of MG against *P. aeruginosa*. MG can interfere with the QS of *C. violaceum* and *P. aeruginosa*, and modulate the occurrence of QS-associated virulence factors, which are crucial to develop new antimicrobials and/or improve the efficacy of existing antimicrobials and to reduce the pathogenicity associated with these bacteria. Moreover, this study determined the molecular mechanism of the QS inhibition of MG in *P. aeruginosa*. MG showed the down-regulation of autoinducer synthase (*lasI* and *rhlI*) and cognate receptor (*lasR* and *rhlR*) genes of *P. aeruginosa* (PAO1). All the findings of this study suggest that methyl gallate can be a safe and potential tool to combat virulence of *C. violaceum* and *P. aeruginosa* in healthcare environment or as a medication to treat *C. violaceum* and *P. aeruginosa* infections in the future.

## Methods

### MIC and MBC Assays

MIC assays were performed according to Clinical and Laboratory Standards Institute (CLSI) guidelines for determining the antimicrobial activity of selected phenolic compounds (MG, pyrogallol, pyrocatechol, resorcinol and phloroglucinol)^82^. Overnight cultures of *C. violaceum* (ATCC12472, ATCC31532 and CV026), and *P. aeruginosa* (PAO1) were grown in lysogeny broth (LB). Cultures were diluted to a final inoculum of 5 × 10^5^ CFU/mL in 96-well microtiter plates, compounds were added with appropriate concentrations and MIC was determined after 18 h incubation at 35 °C. MBCs were determined according to our previously published report^37, 83^. All of the QS assays and anti-virulence assays were performed at concentrations lower than the MIC values known as sub-inhibitory concentrations to ascertain that there was no inhibition.

### Qualitative Anti-QS Activity

Standard disk–diffusion assay was used to detect anti-QS activity of the 5 phenolic compounds utilizing biomonitor strain, *C.violaceum* (ATCC12472) by slightly modifying a previously reported method^84^. *C. violaceum* ATCC12472 was poured on molten LB agar medium in plastic petri dish (90 × 15 mm) to make 5 × 10^6^ CFU/mL. 60 μL of each compounds (15, 30 and 60 μg/60 μL) to be tested were pipetted on sterile paper disks, air dried and placed on solidified agar. They were incubated overnight at 30 °C, and examined for violacein production. QS inhibition was determined by a colorless, opaque, but viable halo around the disks.

### Quantitative Anti-QS Activity

Quantitative evaluation of QS inhibition of phenolic compounds were carried out based on their ability to inhibit the production of purple violacein pigment by *C. violaceum* ATCC12472 according to previously reported methods^85, 86^. The bacterial strain was cultured aerobically in LB at 30 °C with or without the addition of sub-MIC concentrations of phenolic compounds. Furanone (10 μg/mL; Sigma, St. Louis, MO, USA) was used as QSI-positive controls. One milliliter of an overnight culture of *C. violaceum* was centrifuged (13,793 *g*, 10 min) to precipitate the insoluble violacein. The culture supernatant was discarded and the pellet was evenly re-suspended in 1 mL of dimethyl sulfoxide (DMSO). The solution was centrifuged (13,793 *g*, 10 min) to remove the cells and the violacein was quantified at a wavelength of 585 nm using a UV**-**Vis spectrophotometer (UV-1800, Shimadzu, Kyoto, Japan). The percentage of violacein inhibition was calculated by following the formula^85^:

$${\rm{P}}{\rm{e}}{\rm{r}}{\rm{c}}{\rm{e}}{\rm{n}}{\rm{t}}{\rm{a}}{\rm{g}}{\rm{e}}\,{\rm{o}}{\rm{f}}\,{\rm{v}}{\rm{i}}{\rm{o}}{\rm{l}}{\rm{a}}{\rm{c}}{\rm{e}}{\rm{i}}{\rm{n}}\,{\rm{i}}{\rm{n}}{\rm{h}}{\rm{i}}{\rm{b}}{\rm{i}}{\rm{t}}{\rm{i}}{\rm{o}}{\rm{n}}=\frac{{\rm{O}}{\rm{D}}\,{\rm{o}}{\rm{f}}\,{\rm{c}}{\rm{o}}{\rm{n}}{\rm{t}}{\rm{r}}{\rm{o}}{\rm{l}}\,{\rm{a}}{\rm{t}}\,585\,{\rm{n}}{\rm{m}}-{\rm{O}}{\rm{D}}\,{\rm{o}}{\rm{f}}\,{\rm{t}}{\rm{e}}{\rm{s}}{\rm{t}}\,{\rm{s}}{\rm{a}}{\rm{m}}{\rm{p}}{\rm{l}}{\rm{e}}\,{\rm{a}}{\rm{t}}\,585\,{\rm{n}}{\rm{m}}}{{\rm{O}}{\rm{D}}\,{\rm{o}}{\rm{f}}\,{\rm{c}}{\rm{o}}{\rm{n}}{\rm{t}}{\rm{r}}{\rm{o}}{\rm{l}}\,{\rm{a}}{\rm{t}}\,585\,{\rm{n}}{\rm{m}}}\times 100$$

### Effects of MG on the modulation of AHL synthesis and its activity

The agar diffusion double ring assay was performed for qualitative determination of the effect of MG on the inhibition of AHL synthesis (Set-B) and on the modulation of AHL activity (Set-A) using *C. violaceum* (CV) bioassay system (Supplementary Table 1) as described in Supplementary Fig. 4 and in published articles^8, 55, 87^.

In quantitative determination, the effect of MG on AHL synthesis and their activity were determined by slightly modifying a previously reported method^16, 87^. *C. violaceum* ATCC31532 was incubated in the presence of MG (1–16 μg/mL) for 48 h at 32 °C. The cultures were then centrifuged (5,223 *g*, 10 min, 4 °C), and the supernatant was sterile-filtered. The filtrate containing AHL was extracted from the cell free supernatant (5 mL) using dichloromethane (3:1 v/v) and evaporated under vacuum at 45 °C. The isolated AHL was suspended in sterile LB and CV026 was inoculated, and incubated for 24 h at 32 °C. The cultures were subsequently assayed for violacein production as described in “Quantitative Anti-QS Activity” section.

For determining the effect of MG on AHL activity, AHL overproducer strain ATCC31532 was grown for 48 h at 32 °C. The culture was then centrifuged (5,223 *g*, 10 min, 4 °C), and the supernatant was sterile-filtered. The AHL was extracted from filtered supernatant as stated above and supplemented with LB that contained MG (2–32 μg/mL). AHL biosensor strain CV026 was inoculated in AHL containing LB and incubated for 24 h at 32 °C. The bacterial cultures were centrifuged, then the pelleted cells were lysed with 0.1% SDS and the violacein was extracted in DMSO, and the absorbance read as described above.

### Effects of phenolic compounds on swarming motility

The swarming motility of *P. aeruginosa* (PAO1) in presence of phenolic compounds was determined according to a previously described method^37, 47^. LB agar plates containing agar (0.50% w/v) and glucose (0.50% w/v) were used for the motility inhibition assay. Molten agar plates were supplemented with 1/2 × MIC of all these compounds. A non-supplemented drug free-plate was employed as the negative control, and furanone (10 μg/mL) supplemented plate was used as positive control. A single colony of *P. aeruginosa* was inoculated in the center of each plate using a toothpick, and then they were incubated for 16 h at 37 °C. The swarm zone diameters were measured using calibrated digital slide calipers (Mitotoyo, Japan), and photographs of the plates were captured. Molten agar plates were also supplemented with 16, 32, 64, 128, and 256 μg/mL of MG to evaluate the concentration-dependent effects of MG on swarming motility.

### Effects of MG on biofilm formation and viability

The inhibitory effect of MG on biofilm formation was determined by slightly modifying previously reported spectrophotometric methods^88, 89^. Briefly, test compounds were supplemented with trypticase soy broth (TSB) to three separate wells in 96-well microplate for each concentration, so that the final concentration would be 16, 32, 64, 128, and 256 μg/mL of MG after inoculation of bacteria. The 18 h cultures of *P. aeruginosa* incubated at 37 °C with shaking at 200 rpm were diluted with fresh TSB, and added to the designated wells which will be 1 × 10^6^ CFU/mL after inoculation. Optical density at 600 nm was measured immediately after inoculation and after 24 h incubation at 37 °C to monitor planktonic cell growth. To determine the amount of biofilm formation, supernatant from the microplate wells was gently removed and the wells were washed thrice with sterile phosphate buffer saline (PBS, pH 7.2) using a multichannel pipette. The remaining adherent biofilms were fixed by 200 μL of 99% methanol for 20 min, and was then stained with 100 μL of a 0.2% (w/v) crystal violet solution for 15 min at room temperature. The excess stain was removed from the wells by rinsing four times with PBS, and then 100 μL of 95% ethanol was added to extract the crystal violet in solution from the biofilm. The OD of the extracted crystal violet was then measured, yielding a measure of biofilm formation (relative to the control). For optical imaging, crystal violet stained biofilms were washed with sterile PBS and no ethanol extraction was performed. Measurements were performed in triplicate and repeated 3 times.

Previously reported biofilm viability assay methods were utilized to evaluate the effect of MG on the viability of biofilm produced by *P. aeruginosa* ^37, 90^. In brief, 2 mL of the sterile TSB broth was taken in Nunc™ Lab-Tek™ II Chambered Coverglass (ThermoScientific, USA), and *P. aeruginosa* culture was inoculated to the broth to give 1 × 10^6^ CFU/mL. The cultures of *P. aeruginosa* in Nunc™ Lab-Tek™ II Chambered Coverglass were incubated for 48 h at 37 °C to produce biofilms. The broth media was changed every 24 h. After incubation, the Chambered Coverglass were rinsed with 1× PBS, and then 2 mL of sterile TSB containing 16, 32, 64, 128, and 256 μg/mL of MG were added. They were again incubated for 24 h at 37 °C. After 24 h exposure to MG, the biofilms were rinsed with sterile double-distilled water (DDW) and stained with BacLight live/dead stain (ThermoFisher Scientific, MA 02451, USA). Confocal microscopy was used to scan the viable and nonviable biofilms. Imaging was performed in a ZEISS LSM 700 confocal microscope (Zeiss, IL 61801, USA), with 488 nm excitation and 560–600 nm emission range. Both image acquisition and subsequent manipulation were performed using ZEN 2009 software. The untreated biofilm was used as control.

### Effects of MG on elastase activity

Elastase activity was measured by modifying the methods previously described^55^. Briefly, *P. aeruginosa* (PAO1) grown in the presence and absence of MG. One milliliter of 0.5% elastin-congo red solution (in 10 mM PBS) (Sigma Chemical Co., St. Louis, MO, USA) were mixed with 200 μL of *P. aeruginosa* culture supernatant fluids and incubated at 37 °C for 3 h in a water bath. The samples were then vortexed and centrifuged (118 *g*, 10 min, 10 °C) to remove insoluble elastin-congo red. The OD of the supernatant fluids from both the control and treated samples was measured at 494 nm. The percent change in OD was then calculated to determine the decrease in elastase activity. Activity was expressed as change in OD per μg of protein.

### Effects of MG on total proteolytic activity

Total proteolytic activity of the cell-free supernatant fluid of *P. aeruginosa* (PAO1) culture cultivated in the presence and absence of MG was estimated according to a method described earlier^55^. Briefly, 100 μL of culture supernatant fluid was mixed with 900 μL of 0.5% azocasein (Sigma-Aldrich Chemical Co., St. Louis, MO, USA) solution prepared in 50 mM Tris buffer containing 2 mM CaCl_2_. Samples were incubated at 37 °C for 30 min. Then, 15% TCA (300 μL) was added to stop the reaction, and then it was centrifuged (5,223 *g*, 10 min, 4 °C). The OD of the supernatant fluids from both the control and treatment samples was measured at 440 nm. The percent change in OD was then calculated from the OD values.

### Effects of MG on LasA staphylolytic activity

LasA protease activity was determined by measuring the ability of *P. aeruginosa* (PAO1) culture supernatant fluid to lyse boiled *S. aureus* cells^63, 70^. A 30 mL volume of an overnight *S. aureus* culture was boiled for 10 min, and then centrifuged for 10 min at 10,000 *g*. The resulting pellet was re-suspended in 10 mM Na_2_PO_4_ (pH 4.5) to an OD value of approximately 0.8 at 600 nm. A 100 μL aliquot of *P. aeruginosa* LB medium culture supernatant with or without MG was added to 900 μL of a boiled *S. aureus* suspension, and the OD was determined after 0, 5, 10, 15, 20, 30, 45, and 60 min at 600 nm. Activity was expressed as the change in the OD/hour per μg of protein.

### Effects of MG on pyocyanin production

Pyocyanin was extracted from *P. aeruginosa* culture supernatant and measured by the method as described earlier^61^. Briefly, a 5 mL sample of culture grown for 24 h in broth medium containing the test material was centrifuged, and the supernatant extracted with 3 mL of chloroform and then re-extracted into 1 mL of 0.2 M HCl to give a pink to deep red solution. The OD of this solution was measured at 520 nm. Concentrations expressed as pyocyanin produced in μg/mL of culture supernatant were determined by multiplying the OD520 by 17.072.

### Effects of MG on exopolysaccharide production

*P. aeruginosa* (PAO1) grown in the presence and absence of MG were centrifuged and the resulting supernatant was filtered. Three volumes of chilled 100% ethanol were added to the filtered supernatant and incubated overnight at 4 °C to precipitate the exopolysaccharide (EPS)^91, 92^. EPS was then extracted and quantified by measuring sugars as below. A 1 mL aliquot of a carbohydrate solution is mixed with 0.5 mL of 5% aqueous solution of phenol in a test tube. Subsequently, 2.5 mL of concentrated sulfuric acid added rapidly to the mixture. After allowing the test tubes to stand for 10 min, they are vortexed for 30 s and placed for 20 min in a water bath at room temperature for color development. Then, OD at 490 nm is recorded on a spectrophotometer. Reference solutions are prepared in identical manner as above, except that the 1 mL aliquot of carbohydrate is replaced by distilled water.

### Effects of MG on rhamnolipid production

One of the most widely used methods for ramnolipid quantification is the orcinol test^93, 94^. Briefly, 300 μL supernatant from PPB (containing 2% glycerol) culture was extracted twice using 600 μL diethyl ether. The ether layer was transferred to a new tube and allowed to evaporate. Residues were dissolved in a solution of 100 μL H_2_O, 100 μL 1.6% orcinol (Sigma Chemical Co., St. Louis, MO, USA), and 800 μL 60% H_2_SO_4_. After heating for 30 min at 80 °C, tubes were cooled at room temperature for 15 min, and the OD was measured at 421 nm. Rhamnose concentrations were determined using the standard curve equation, y = 0.0033x − 0.0077 and concentrations of rhamnolipids were calculated by multiplying rhamnose values by a coefficient of 2.5, as previously described^66^.

### Effects of MG on the expression of quorum sensing genes

*P. aeruginosa* (PAO1) was treated with 16, 32, 64, 128, and 256 μg/mL of MG for 16 h with the supplementation of 100 μM autoinducer (3-oxo-C_12_-HSL), and total RNA was extracted according to the protocol for TRIZOL reagent (Sigma). The purity of RNA was confirmed and concentration (μg/mL) was calculated by measuring OD at 260 and 280 nm using a U-2800 spectrophotometer (Hitachi High Technologies, Tokyo, Japan). cDNA was synthesized from 100 ng of RNA by using RNA to cDNA EcoDry Premix (Oligo dT) (Clontech Laboratories, Inc., Seoul 153–779, Republic of Korea) according to the supplied protocol. The expressions of target genes were detected by performing real-time PCR with the primers listed in Supplementary Table 2. To each PCR tubes (TLS0851, Bio-Rad Laboratories Inc., Herts HP27DX, United Kingdom), 12.5 μL of SYBR Select Master Mix for CFX (Applied Biosystems, Foster City, CA 94404, USA), 1 μL of forward primer (10 pmol) and 1 μL of reverse primer (10 pmol) of target gene, 9.5 μL of RNase-free water and 1 μL of cDNA were added, mixed by pipetting and spined down. The real time PCR reaction was accomplished in CFX96 Touch™ Real-Time PCR Detection System (Bio-Rad Laboratories Inc., Irvine, CA 92618, USA) with 40 cycles of denaturation at 95 °C for 15 s, annealing at 60 °C for 15 s and elongation at 72 °C for 30 s. mRNA expressions were normalized using β-actin. All mRNA expressions were expressed in relation to the average expression of the non-treated group (100%).

### Cell viability assay

The *in vitro* cytotoxicity of MG was determined by means of reported methods of MTT assay with slight modification^95, 96^. In brief, the RAW 264.7 macrophage cells (Korean Cell Line Bank, Seoul, Republic of Korea) were cultured in 100 μg/mL of streptomycin, 100 IU/mL penicillin, and 10% fetal bovine serum (FBS)-supplemented RPMI 1640 medium, at 37 °C under a humidified atmosphere of 5% carbon dioxide (CO_2_). The cells (2 × 10^4^ cells/mL) were acclimated for 24 h onto 96-well plates containing 100 μL of RPMI medium with the added substances mentioned above. Afterward, the cells were treated with 0, 0.78, 1.56, 3.13, 6.25, 12.50 and 25.00 mg/mL of MG. Twenty microliter of MTT solution (5 mg/mL) was added in each well after 24 h of treatment, and incubated again for 4 h. Then, the supernatant was discarded, and DMSO (200 μL) was supplemented to each well. The cells in DMSO were placed at room temperature for 30 min to completely solubilize the formazan crystals. The optical density was measured at 570 nm. The cells not treated with any drugs were assigned as control. The cell viability (%) was calculated by the following formula and the inhibitory concentration fifty percent (IC_50_) of MG was determined by using Graphpad prism 5.0 (California, USA).

Viable Cells (%) = (OD of drug-treated sample/OD of non-treated sample) × 100, where OD is the optical density^97^.

### Statistical analysis

Data are represented as mean ± standard deviation (SD) of triplicate assays. SAS software (SAS Institute Inc., Cary, NC, USA) was utilized for statistical analysis. Values were appraised by one way analysis of variance (ANOVA) following F-test. *P*-values of less than 0.05 were regarded as statistical significant which are illustrated by different alphabets.

## Electronic supplementary material

Supplementary information 
