# Supplementary material for: Impact of phenolic compounds in the acyl homoserine lactone-mediated quorum sensing regulatory pathways
Source: Sci Rep. 2017 Sep 6;7:10618. doi: 10.1038/s41598-017-10997-5 (PMC5587592; doi:10.1038/s41598-017-10997-5)
Supplement: Supplementary file 1 — Supplementary information [file 41598_2017_10997_MOESM1_ESM.pdf]

Supplementary Information

Impact of phenolic compounds in the acyl homoserine lactone-mediated quorum sensing regulatory pathways

Md Akil Hossain[1,2], Seung-Jin Lee[1], Na-Hye Park[1], Abraham Fikru Mechesso[1], Biruk Tesfaye Birhanu[1], JeongWoo Kang[2], Md Ahsanur Reza[3], Joo-Won Suh[4,*] & Seung-Chun Park[1,*]

[1]Laboratory of Clinical Pharmacokinetics and Pharmacodynamics, College of Veterinary Medicine, Kyungpook National University, Daegu 702-701, Republic of Korea.

[2]Veterinary drugs & Biologics Division, Animal and Plant Quarantine Agency (QIA), 177, Hyeoksin 8-ro, Gimcheon-si, Gyeongsangbuk-do 39660, Republic of Korea.

[3]Faculty of Animal Science and Veterinary Medicine, Patuakhali Science and Technology University (Outer Campus), Babugonj, Barisal-8210, Bangladesh.

[4]Center for Nutraceutical and Pharmaceutical Materials, Division of Bioscience and Bioinformatics, Science campus, Myongji University, 449-728, Yongin, Gyeonggi, Republic of Korea.

[*]Correspondence and requests for materials should be addressed to S.C.P. (parksch@knu.ac.kr) & J.W.S (jwsuh@mju.ac.kr).

Supplementary Materials and Methods:

Existence of methyl gallate in ethyl acetate fraction of *Nymphaea tetragona* 50% methanol extract (NTME). The compounds in the extract were identified by Liquid chromatography-massspectrometry (LC-MS) analysis. Agilent series 1100 HPLC instrument (Agilent, Waldbronn, Germany) equipped with a quaternary pump, a diode-array detector (DAD), an autosampler, and a column compartment. LC-MS analysis was carried out by an Agilent triple quadrupole mass spectrometer (MS, 6400) assembled with the Agilent Liquid chromatography (LC) system. The compounds were separated on a Zorbax Eclipse XDB-C18 column (4.6 × 150 mm, 5 μm). The mobile phase is the mixture of acetonitrile (A), and 0.1% formic acid (B) with the gradient flow as: 10% of A (0–5 min), 25% of A (5.1–8 min), 35% of A (8.1–15 min), 60% of A (15.1– 17 min), 35% of A (17.1–20 min) and, 10% of A (20.1–28 min). The flow rate was 0.8 mL/min, and the temperature of the column was 30°C. The DAD was monitored at 360 nm, and the on-line UV spectra were recorded in the range 190–400 nm. Compounds were analyzed in negative ion mode.

HPLC analysis of NTME was performed to determine whether the purchased pure methyl gallate is same as identified in NTME or not. A published reverse-phase HPLC method was optimized for the better identification and quantification of methyl gallate[1]. Hewlett-Packard Agilent 1100 HPLC system (Agilent Technologies, Santa Clara, CA 95051, USA) equipped with a G1322A degasser, a G1311A quaternary pump, a G1313A auto-sampler, a G1316A column compartment, and a G1314A UV detector was used. Agilent ChemStation software (Agilent Technologies, Santa Clara, CA 95051, USA) was used to perform the chromatographic analysis. The 5 μm C18 column with, 150 mm length and 4.6 mm inner diameter (Fortis Technologies Ltd, Cheshire, CH64 3UG, United Kingdom) was

used for the separation of compounds. The mobile phase was a mixture of methanol and 0.1% phosphoric acid with a ratio of 70 and 30 parts respectively. The flow rate of the mobile phase was 1 mL/min and the column compartment was maintained at 25℃. The injection volume of the filtered pure compound and extract solution were 20 µL. The UV detection of the compound was done at a wavelength of 279 nm. The run time for one sample was 10 min.

Supplementary Table 1. Description of bacterial strains used for quorum sensing inhibition bioassay.

**Bacterial strains**	**Description**	**Purpose**	**Source or reference**
Chromobacterium violaceum ATCC12472	Type strain	QSI indicator strain	ATCC
Chromobacterium violaceum ATCC31532	Non-pigmented, C_6 HSL overproducer	Positive control for AHL assay	ATCC
Chromobacterium violaceum CV026	Mini Tn5 mutant of ATCC31532 and Detects C_4 - C_6 HSLs	AHL biosensor	[2]
Pseudomonas aeruginosa PAO1	C_4 and 3-oxo-C_{12} HSL producer	Positive control for QSI	V. Deretic

QSI, quorum sensing inhibitor; HSL, homoserine lactones; AHL, acyl homoserine lactone.

Sources: ATCC, American Type Culture Collection; V. Deretic, Department Molecular Genetics and Microbiology, University of New Mexico Health Sciences Center, Albuquerque, NM, USA.

Supplementary Table 2. List of primers used in this study with their sequences and amplicon sizes.

Primers	Sequence	Amplicon size (BP)
lasI	F 5′-GCCCCTACATGCTGAAGAACA-3′	62
	R 5′-CGAGCAAGGCGCTTCCT-3′	
lasR	F 5′-GTGGAGCGCCATCCTGCAGA-3′	144
	R 5′-CGGTCGTAATGCTCGCGCCA-3′	
rhlI	F 5′-AGCTGGGACGCTACCGGCAT-3′	136
	R 5′-TGGCGGCTCATGGCGACGAT-3′	
rhlR	F 5′-GAGGAATGACGGAGGCTTTTTG-3′	255
	R 5′-CTTCTTCTGGATGTTCTTGTGG-3′	
pqsA	F 5'-CCACTCCGCTGGACGACAAC-3'	84
	R 5'-GCAGCATGTGCGAGGGAATC-3'	
rpoD	F 5′-GGGCGAAGAAGGAAATGGTC-3′	178
	R 5′-CAGGTGGCGTAGGTGGAGAA-3′	

Supplementary Results:

Confirmation of compound profile of *Nymphaea tetragona* 50% methanol extract. The LC-MS analysis showed that the extract contains gallate, methyl gallate, and some unidentified compounds (Supplementary Figure 1A). The retention times, detection wavelength (λ_{max}), and mass to charge ratio (*m/z* $(M\text{-}H)^-$) were 1.40 min, 272 nm and 169, respectively for gallate, and 4.17 min, 274 nm and 183, respectively for methyl gallate. Two other intense peaks were found with 951 and 633 mass to charge ratio (*m/z* $(M\text{-}H)^-$); 271 and 266 nm of wavelength at a retention time of 4.81 and 4.91 min, but were not identified.

Fingerprint analysis by HPLC assay revealed the similarity of methyl gallate that we identified in NTME with that we used in the current study. The method for the determination of methyl gallate was optimized and validated according to the standard guidelines. The linearity was achieved in a concentration range from 5 µg/mL to 80 µg/mL. The equation of standard curve was $y = 69.197x – 40.942$, and the regression value (R^2) was 0.9998. A peak of methyl gallate with good quality was identified from the solution of pure methyl gallate at about 7.5 min. Similarly, the analysis of ethyl acetate fraction (EAF) of NTME gave the peak of methyl gallate at the same time point. The area of the methyl gallate peak from ethyl acetate fraction of NTME is smaller than the peak from pure compound. Representative chromatograms of pure methyl gallate (A), and EAF of NTME (B) is presented in Supplementary Fig. 1.

Supplementary Fig. 1: Confirmation of compound profile of *Nymphaea tetragona* 50% methanol extract. (A) Representative PDA chromatogram and selected ion mass chromatograms of *Nymphaea tetragona* 50% methanol extract. Peaks with retention time of 1.40 and 4.17 min are gallate, methyl gallate, respectively. (B) Representative chromatograms of pure methyl gallate (a), and EAF of NTME (b) from HPLC fingerprint analysis.

A

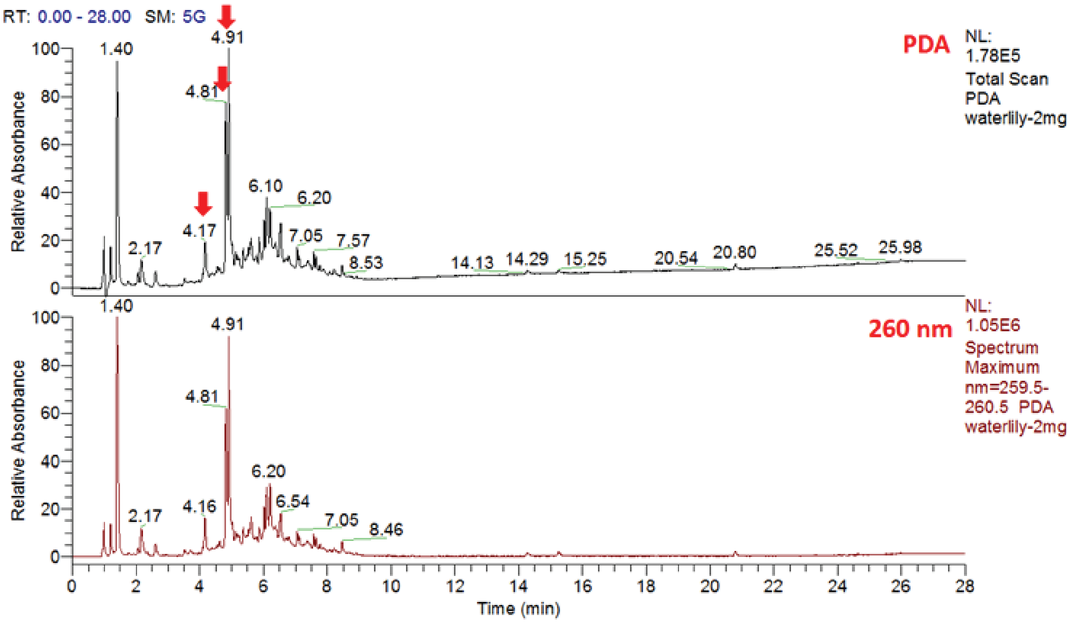

B

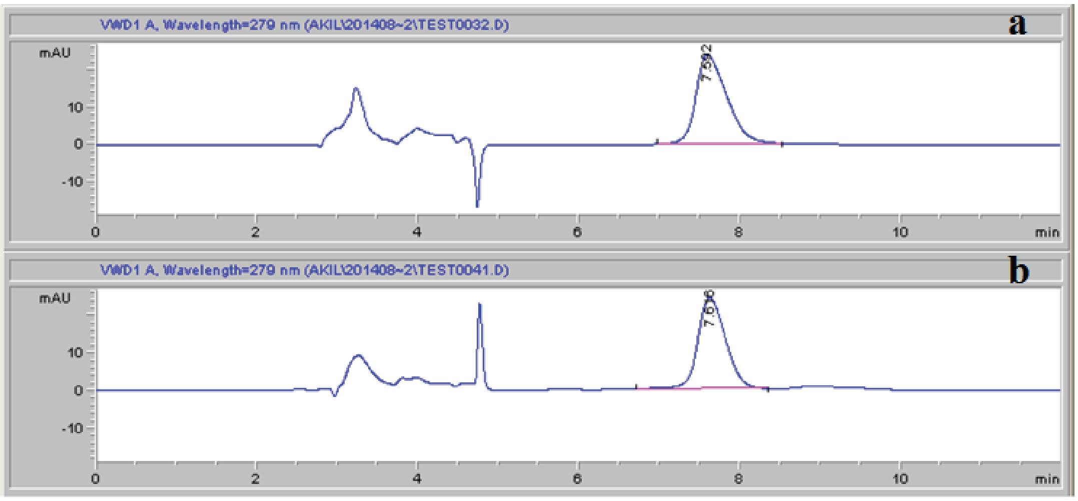

Supplementary Fig. 2: Qualitative quorum sensing inhibition of methyl gallate. Methyl gallate (A) 60 µg/disk, (B) 30 µg/disk, (C) 15 µg/disk, and (D) Furanone 10 µg/disk, (E) Tetracycline 10 µg/disk, (F) Sterile doubled-distilled water.

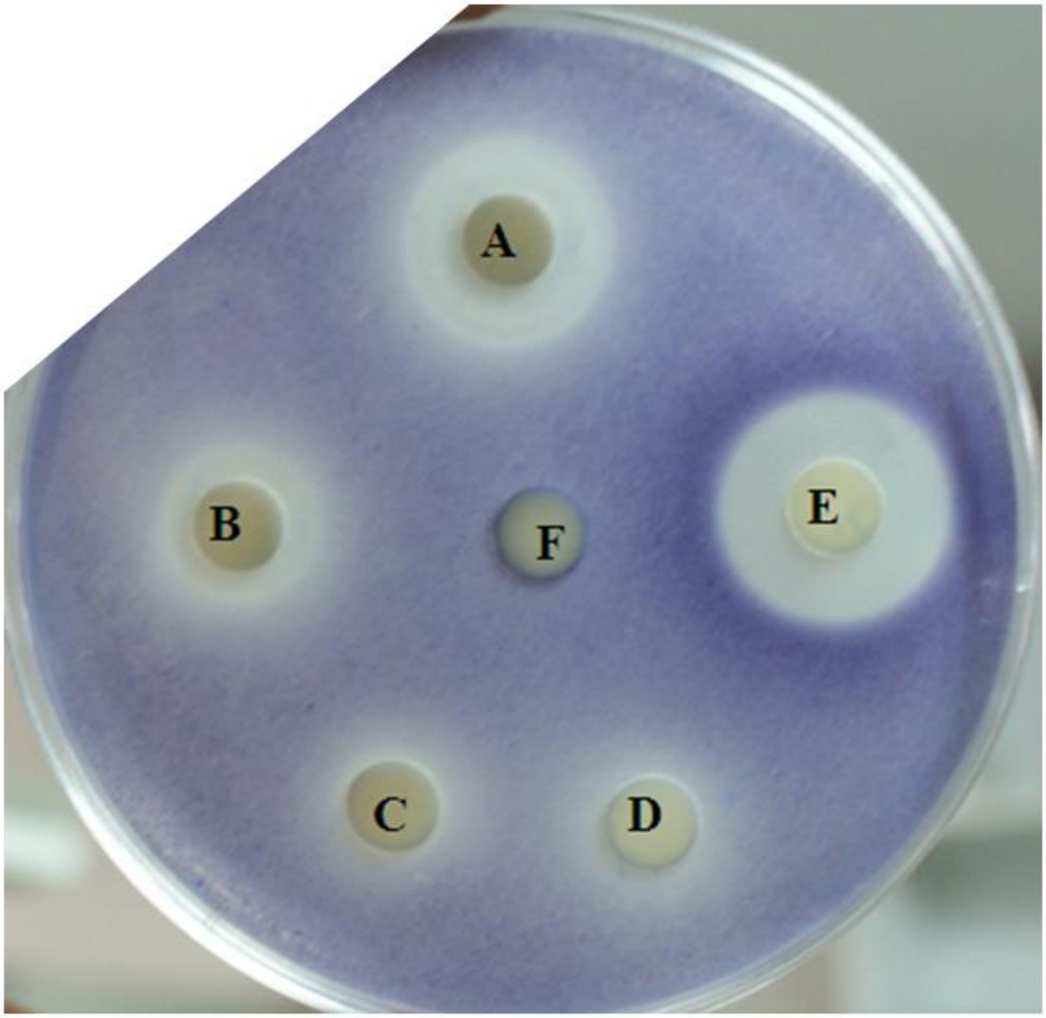

Supplementary Fig. 3: A qualitative determination of the effect of methyl gallate on AHL synthesis and their activity. Effect on (A) AHL synthesis, and (B) AHL activity.

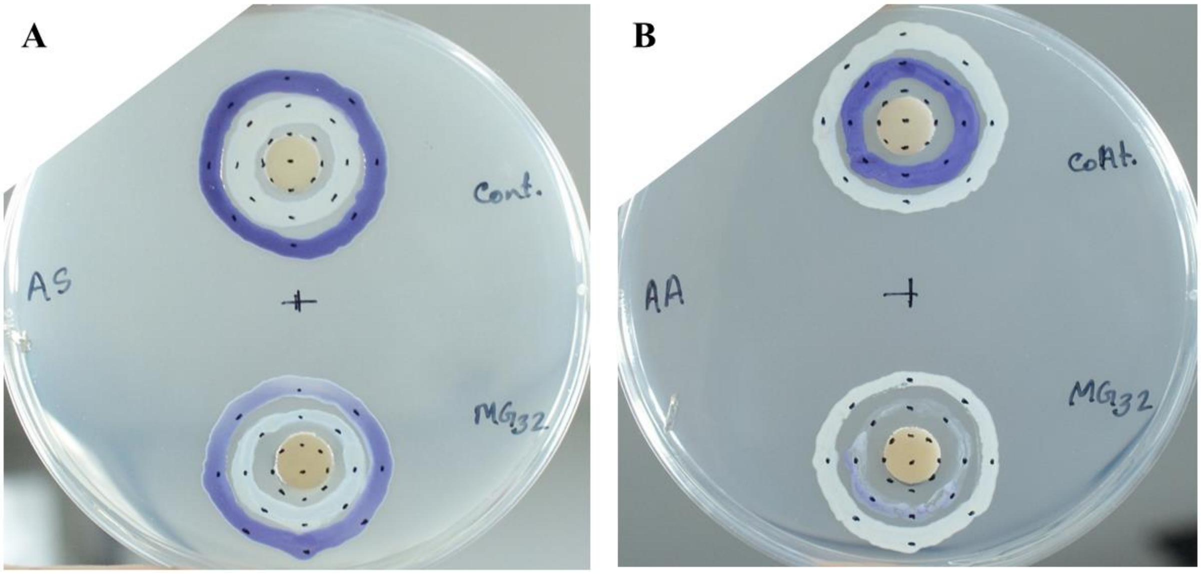

Supplementary Fig. 4: A schematic representation of qualitative agar diffusion assay for quorum sensing inhibition. Petri dish (A) represents the inhibition of AHL activity and petri dish (B) represents the inhibition of AHL synthesis[2].

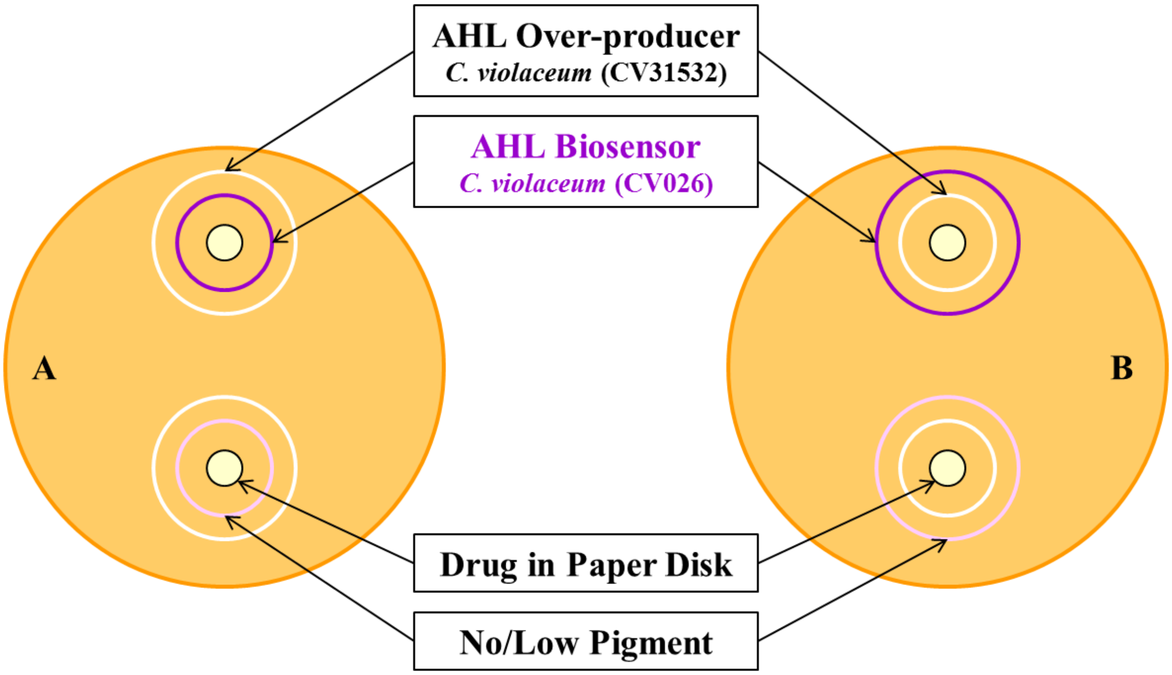

Supplementary Fig. 5: The optimization of incubation time and the amount of autoinducer for better expression of *lasR* genes in *P. aeruginosa* (PAO1). (A) Represents optimization of incubation time, and (B) represents the optimization of the amount of autoinducer (3O-C12-HSL) need to supplement for better expression of QS genes. Data represent the mean±SD of triplicate analyses. Different superscript letters indicate significant differences at $P < 0.05$.

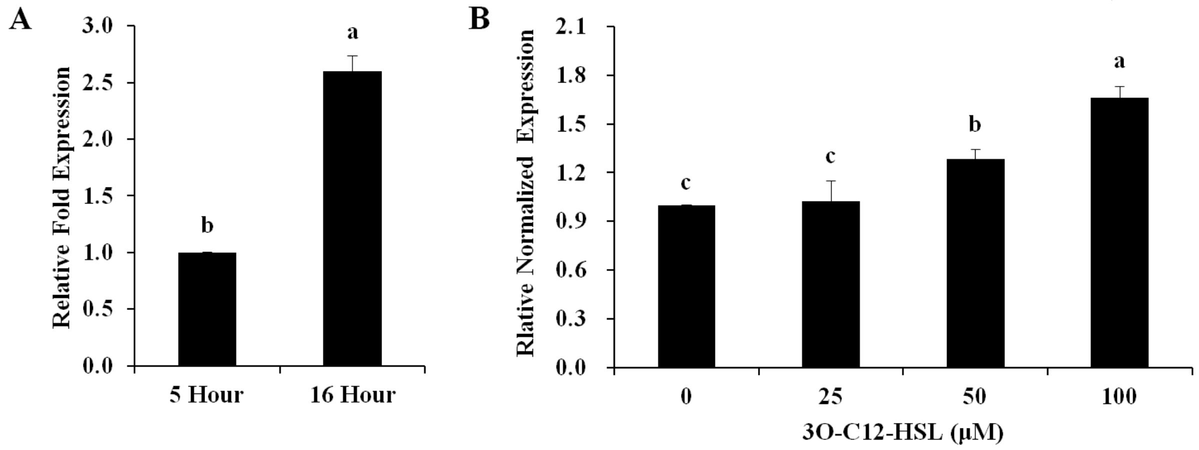

Supplementary References:

1. Dong-mei, W., Jia-hong, C., Yong-mei, W., Liang-liang, Z. & Man, X. Quantitative determination of methyl gallate by HPLC method. *Biomass Chem Eng* **44,** 6–9 (2010).
2. McClean, K.H. *et al*. Quorum sensing and *Chromobacterium violaceum*: Exploitation of violacein production and inhibition for the detection of N-acyl homoserine lactones. *Microbiology* **143,** 3703–3711 (1997).
